# Imbalanced NK cell subpopulations and TIGIT expression limit cetuximab efficacy in colorectal cancer: A promising target for treatment enhancement

**DOI:** 10.1002/ctm2.70351

**Published:** 2025-06-09

**Authors:** Carmen Navarrete‐Sirvent, Ana Mantrana, Aurora Rivas‐Crespo, Alejandra Díaz‐Chacón, Nerea M. Herrera‐Casanova, María Teresa Sánchez‐Montero, Marta Toledano‐Fonseca, María Victoria García‐Ortiz, María José Ortiz‐Morales, Gema Pulido, Mª Teresa Cano, Auxiliadora Gómez‐España, Rafael González‐Fernández, Lionel Le Bourhis, Silvia Guil‐Luna, Antonio Rodríguez‐Ariza, Enrique Aranda

**Affiliations:** ^1^ University of Córdoba/Maimónides Biomedical Research Institute of Córdoba (IMIBIC) Córdoba Spain; ^2^ Cancer Network Biomedical Research Centre (CIBERONC) Madrid Spain; ^3^ Centro Nacional de Investigaciones Oncológicas (CNIO) Madrid Spain; ^4^ Department of Medicine, Faculty of Medicine University of Córdoba Córdoba Spain; ^5^ Medical Oncology Department Reina Sofía University Hospital Córdoba Spain; ^6^ Immunology Department Reina Sofía University Hospital Córdoba Spain; ^7^ Université Paris Cité, INSERM U1160, Institut de Recherche Saint‐Louis Paris France; ^8^ Veterinary Pathological Anatomy Department University of Córdoba Córdoba Spain

1

Dear Editor,

Antibody‐dependent cell‐mediated cytotoxicity (ADCC) induced by natural killer (NK) cells is one important mechanism by which the anti‐EGFR cetuximab exerts its anti‐tumoral effect,^1^ but no studies have examined NK cell profiles as a predictor of response to cetuximab in metastatic colorectal cancer (mCRC). Our findings suggest that imbalanced NK cell subpopulations and the expression of inhibitory receptors on NKs are key mechanisms that may limit the effectiveness of cetuximab, highlighting TIGIT as a promising target for enhancing treatment outcomes.

We collected blood samples from 31 mCRC patients prior to cetuximab treatment to purify NK cells for immune profiling and proteomic analyses. Patients were categorized as non‐responders (NR, *n* = 14) if they progressed before 9 months, and responders (R, *n* = 17) if progression occurred after (Appendix 1). Results of clinical‐pathological data and survival analyses are shown in Appendix 2 (Tables S1, S2). The only significant clinical parameter distinguishing NR and R patients was the number of cetuximab cycles (Appendix 2, Table S1). In terms of survival (Appendix 3, Figure S1), NR patients showed shorter overall survival (OS) and time to progression (TTP) compared with R patients. Besides, in our cohort the median OS and TTP were 21 and 9 months, respectively, which are comparable to results from clinical trials such as COIN (OS: 17 months, TTP: 8.6 months) or TAILOR (OS: 20 months and TTP: 9 months).^2^ These findings validate the clinical relevance of our cohort and underscore the need for biomarkers to aid in the selection of patients who would benefit from cetuximab treatment.

To compare the molecular phenotype of NK cells between R and NR patients before cetuximab treatment, we analyzed their proteomic profiles using a data‐independent acquisition (DIA) approach. Of the 4914 proteins identified, 932 were differentially expressed, with 585 downregulated and 347 upregulated in NR compared with R patients (Figure 1A; Appendix 2: Tables S3, S4). PCA analysis (Figure 1B) and unsupervised clustering (Figure 1C) revealed distinct protein expression patterns that clearly distinguished between the two groups. Moreover, the GSEA analysis of proteomic data (Figure 1D,E) revealed alterations in several biological processes in NKs from NR, including TGFβ, p53, TNFα, and KRAS signalling pathways, suggesting NK dysfunction.^3^, ^4^ Hence, metabolic alteration in NK cells has been linked to the overexpression of peroxisome proliferator‐activated receptors (PPARs), which suppresses mTOR‐mediated glycolysis and interferes with the maturation process from less cytotoxic to potent cytotoxic NK phenotypes.^5^ Therefore, the overactivation of these pathways may contribute to impaired NK function through a variety of immune evasion mechanisms which correlates with the reduced responsiveness of NR patients to cetuximab treatment. These findings support the role of NK cell regulation and phenotype as key factors in the cetuximab response in mCRC.

**FIGURE 1:**
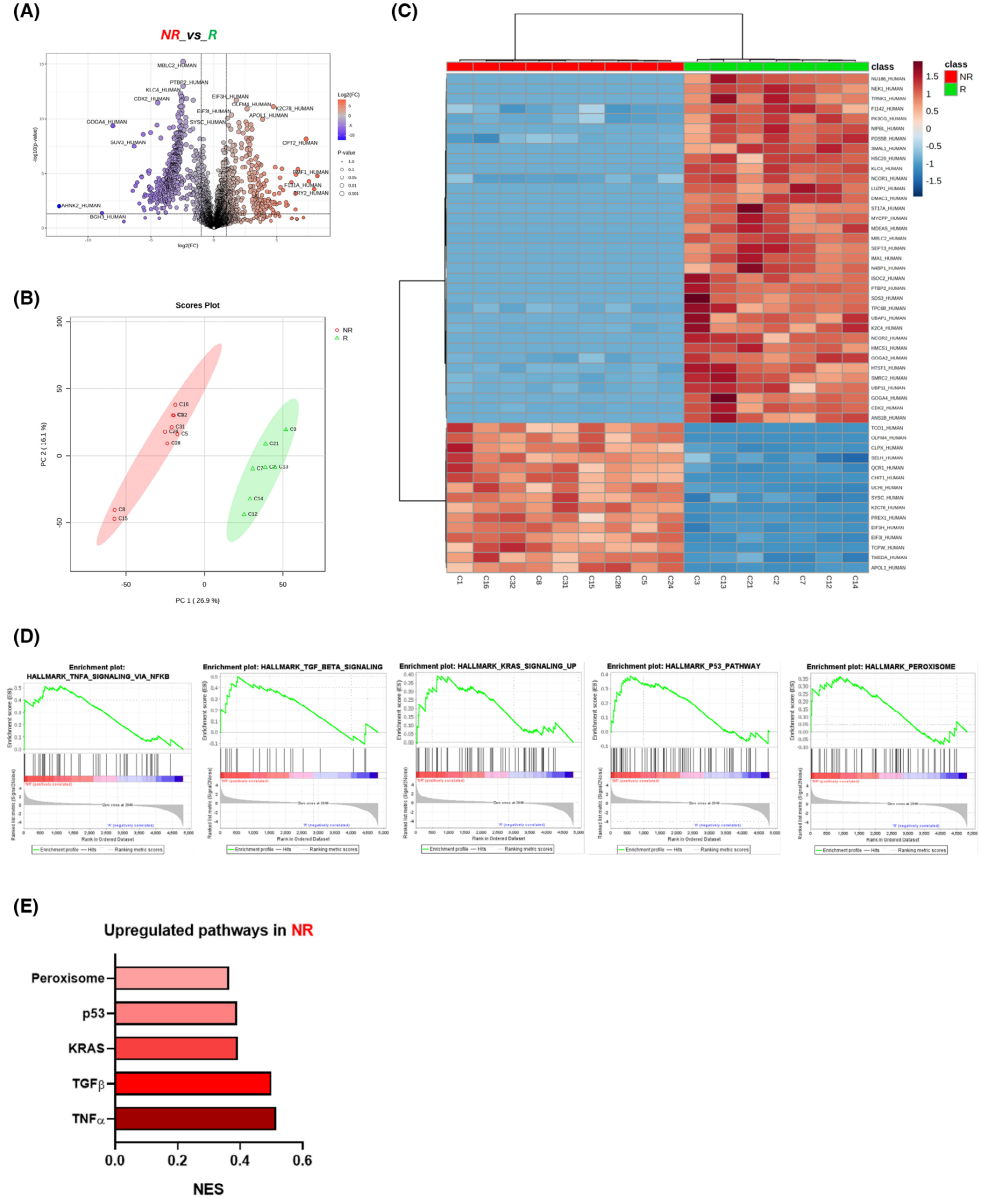
Proteomic analysis reveals dysregulated molecular pathways in NK cells from cetuximab non‐responders’ patients. Distinct proteomic signatures in NK cells from non‐responders (NR) and responders (R) patients to cetuximab were identified using a proteomic approach. (A) Volcano plot with 932 proteins differentially expressed (585 downregulated and 347 upregulated) when comparing NR to R patients, using fold‐change > 2 and *p*‐value < .05 as thresholds. (B) Principal components analysis (PCA) of samples from R and NR patients. (C) Heatmap with the unsupervised clustering NR and R patients using top 50 significant proteins. (D) GSEA plots of significantly upregulated gene pathways in NR patients (E) and their corresponding normalized enrichment score (NES).

Notably, and consistent with the proteomic analysis, although no differences were observed in CD45+ lymphocytes (Appendix 3: Figure S2) NK cell immunophenotyping data (Figure 2) confirmed that NR patients exhibited a higher percentage of non‐cytotoxic NKs (CD56^Bright^ and CD56^Dim^ CD16^−^) and elevated expression of CD57 and the inhibitory receptor TIGIT, combined with reduced levels of cytotoxic and activating receptors, including NKG2A, NKG2C, NKp30, and NKp46. Similar results were found when these markers were analysed in NKT cells (Appendix 3: Figure S3). This profile strongly indicates a senescent or exhausted NK cell phenotype in NR patients, which may underlie their lack of responsiveness to cetuximab.^6^

**FIGURE 2:**
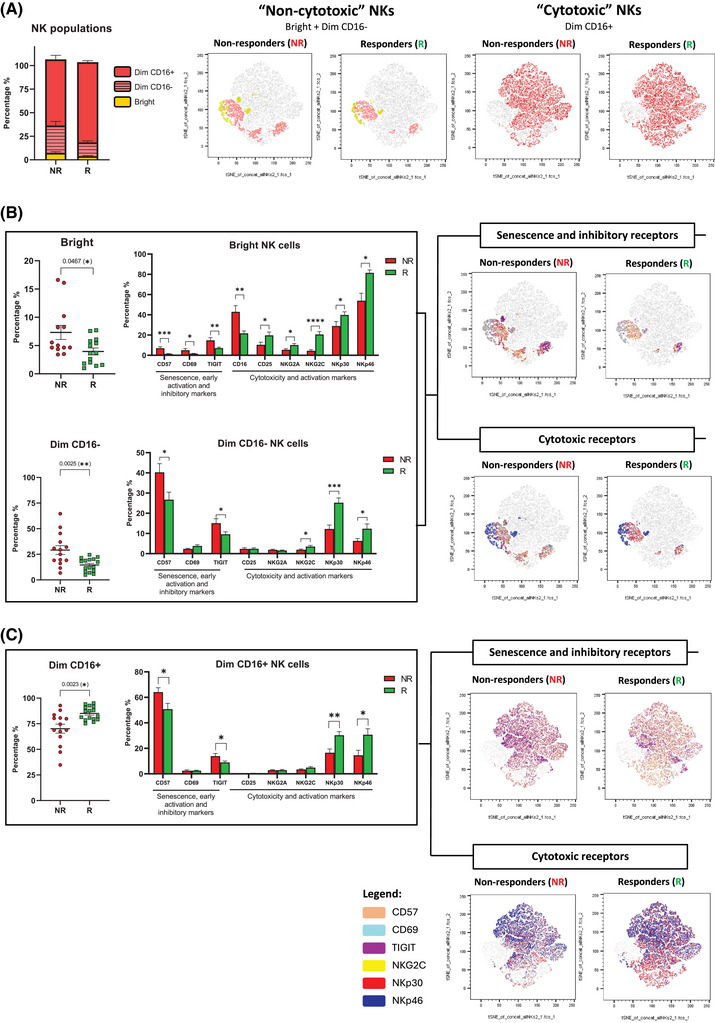
NK cells from cetuximab non‐responding patients exhibit a distinct immunophenotype. (A) NK cell populations analysed and tSNE plots of “non‐cytotoxic” NKs (Bright and Dim CD16−) and “cytotoxic” NKs (Dim CD16+). (B) Bar graphs showing the most significative markers in “non‐cytotoxic” NKs and tSNE plots and (C) in “cytotoxic” NKs. Data are shown as mean ± SEM and statistical significance is indicated as *p‐value < .05; **p < .01; ***p < .001; ****p < .0001.

To further assess the potential of these receptors as predictive biomarkers for cetuximab response, we constructed ROC curves, which revealed that these NK cell‐related parameters have strong predictive value (Appendix 3: Figure S4A). Among these biomarkers, TIGIT proved to be highly effective, achieving an area under the curve (AUC) of .8410, which indicates its high predictive value, and reinforces the association between high TIGIT expression and non‐response to treatment.

TIGIT, also known as WUCAM, VSTM3, and VSIG9, has been identified as an inhibitory receptor in T cells, NK cells, memory T cells, and Tregs.^7^ Remarkably, TIGIT has significant implications for tumour progression and immune evasion.^8^ However, to date few studies have related circulating TIGIT+ immune cells with poor prognosis in cancer. In our study, higher expression of TIGIT in circulating NK cells was associated with poor outcomes in mCRC patients receiving cetuximab as first‐line treatment (Appendix 3: Figure S4B,C). Therefore, TIGIT expression on NK cells emerges as a valuable clinical marker for predicting treatment outcomes in mCRC patients undergoing cetuximab as first‐line therapy.

Our findings of elevated TIGIT expression in NK cells from NR patients strongly support TIGIT as a promising target for combination cancer immunotherapy strategies aimed at enhancing ADCC efficacy. To date, no studies have explored this therapeutic approach. Our in vitro experiments demonstrated that TIGIT blockade with tiragolumab, in combination with cetuximab, not only boosted cetuximab‐mediated ADCC (Figure 3; control: 12.77%, cetuximab: 24.90% and combined treatment: 42.7%) but also significantly facilitated the infiltration and activation of NKs within tumour spheroids (Figure 4; increment control:1, cetuximab: 1.226 and combined treatment: 1.683). These findings are in line with studies demonstrating that TIGIT blockade can promote increased NK cell infiltration and improved function within tumours.^9^ Additionally, our data, along with findings from other studies, indicate that TIGIT expression is higher on CD56^Dim^ NK cells,^10^ which could explain the differential effects of TIGIT blockade observed across NK cell subsets.

**FIGURE 3:**
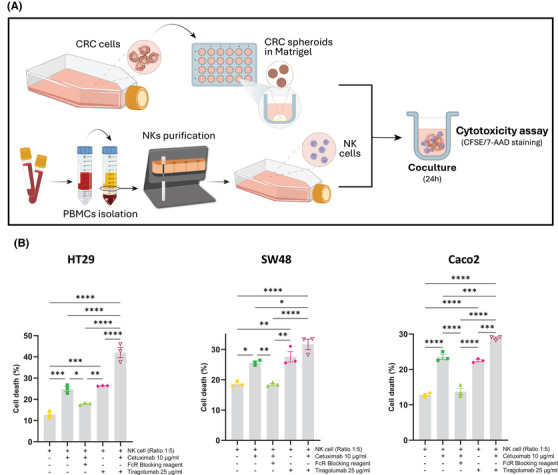
Anti‐TIGIT therapy enhances NK cell efficacy in cetuximab‐mediated ADCC. (A) HT29, Caco‐2 and SW48 cancer cell spheroids were generated and then co‐cultured with activated NK cells from healthy donors in the presence of cetuximab and/or tiragolumab NK cell‐mediated ADCC in co‐cultures was evaluated using the 7‐AAD/CFSE cell‐mediated cytotoxicity assay kit (figures made in Adobe Illustrator). (B) Data are mean ± SEM (*n* = 3) and statistical significance is indicated as *p*‐value. *p‐value < .05; **p < .01; ***p < .001; ****p < .0001.

**FIGURE 4:**
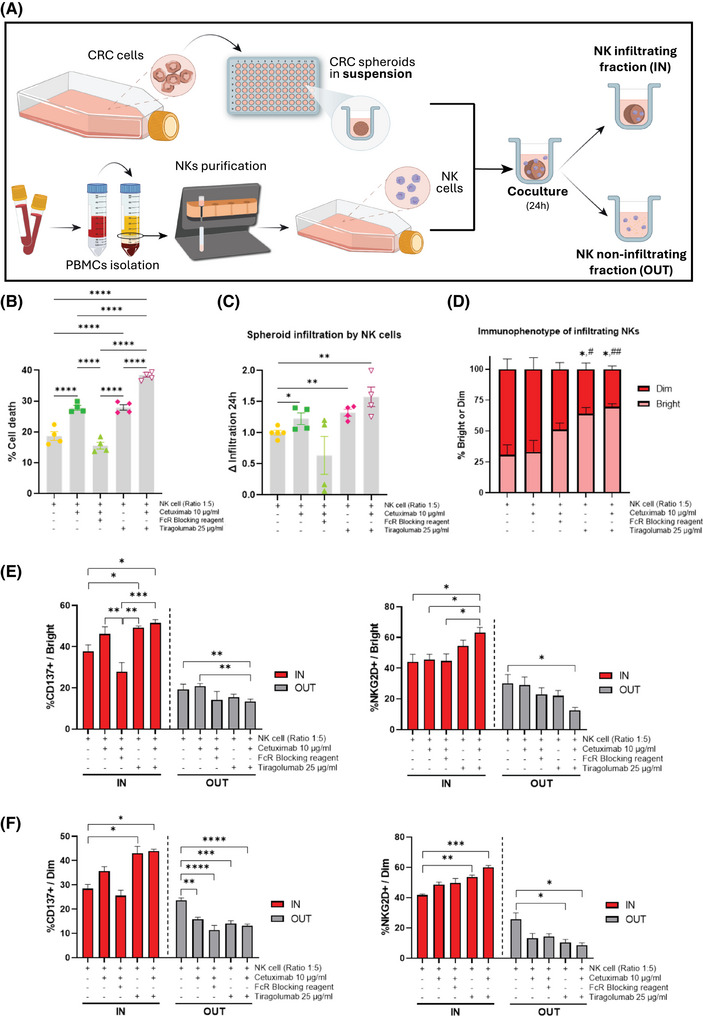
Infiltration assays using the IN/OUT technique. (A) Scheme of generation of spheroids derived from HT29 cell line and coculture IN/OUT protocol (figures made in Adobe Illustrator). (B) Cytotoxicity results using Annexin V/DAPI for cell death analysis. (C) Increment of spheroid infiltration in 24 h. (D) Activation markers in IN and OUT fraction in bright and (E) dim NK cell subsets (*: vs. Control; #: vs cetuximab group). *p‐value < .05; **p < .01; ***p < .001; ****p < .0001. Four independent experiments with four different donors were performed.

In conclusion, our findings suggest that the imbalance of NK cell subpopulations, along with the expression of inhibitory receptors on NK cells, plays a crucial role in limiting the effectiveness of cetuximab in mCRC patients. Specifically, the presence of inhibitory receptors like TIGIT on NK cells appears to dampen their ability to mediate anti‐tumour responses, thereby reducing the therapeutic efficacy of cetuximab. These results underscore the importance of targeting these inhibitory pathways to enhance the function of NK cells within the tumour microenvironment. In particular, TIGIT emerges as a promising target for improving treatment outcomes and boosting the effectiveness of cetuximab‐based therapies in mCRC. Further validation in larger prospective studies is needed to confirm the clinical utility of TIGIT and to guide patient selection.

## AUTHOR CONTRIBUTIONS

Carmen Navarrete‐Sirvent: Investigation, methodology, and writing‐original draft. Ana Mantrana: Investigation and methodology. Aurora Rivas‐Crespo: Investigation. Alejandra Díaz‐Chacon: Investigation. Nerea M. Herrera‐Casanova: Formal analysis. María Teresa Sánchez‐Montero: Investigation. Marta Toledano‐Fonseca: Investigation. María Victoria García‐Ortiz: Investigation. María José Ortiz‐Morales: Resources. Gema Pulido: Resources. Mª Teresa Cano: Resources. Auxiliadora Gómez‐España: Resources. Rafael González‐Fernández: Resources and methodology. Lionel Le Bourhis: Methodology and writing—review & editing. Silvia Guil‐Luna: Methodology and writing—review & editing. Antonio Rodriguez‐Ariza: Conceptualization, writing—review & editing, and supervision. Enrique Aranda: Conceptualization, writing—review & editing, and supervision

## CONFLICT OF INTEREST STATEMENT

The authors declare no conflict of interest.

## FUNDING INFORMATION

Instituto de Salud Carlos III (ISCIII), “PI20/00997”, co‐funded by the European Union. A.R.A. was funded with a researcher contract through the program “Nicolás Monardes” from Junta de Andalucía.

## DATA AVAILAIBILITY STATEMENT

The authors confirm that the data supporting the findings of this study are available in the PRIDE database with the project accession ID PXD057989.

## ETHICS STATEMENT

The study protocol was approved by the Ethics Committee of the Reina Sofia Hospital (COLO‐NK, Committee Reference 4884, version 1.0—01/12/2020) in accordance with the World Medical Association Code of Ethics (Declaration of Helsinki, 2017). Informed consent was obtained from all the patients prior to their enrolment in the study.

## CONSENT FOR PUBLICATION

Not applicable.

## Supporting information



Supporting Information

Supporting Information

Supporting Information
