# Supplementary material for: Imbalanced NK cell subpopulations and TIGIT expression limit cetuximab efficacy in colorectal cancer: A promising target for treatment enhancement
Source: Clin Transl Med. 2025 Jun 9;15(6):e70351. doi: 10.1002/ctm2.70351 (PMC12148951; doi:10.1002/ctm2.70351)
Supplement: Supplementary file 1 — Supporting Information [file CTM2-15-e70351-s001.docx]

**Appendix 1: Supplementary methods**

**Índex**

[1. Patients and clinical-pathological data collection 1](#_Toc187321900)

[2. Immunophenotyping of Peripheral Blood Mononuclear Cells (PBMCs) and NK cell purification 2](#_Toc187321901)

[3. Data-independent acquisition (DIA) proteomics analysis 4](#_Toc187321902)

[4. Human CRC cell lines 5](#_Toc187321903)

[5. Generation of three-Dimensional (3D) tumour spheroids for cytotoxicity assays 6](#_Toc187321904)

[6. Tumour spheroids infiltration assays 7](#_Toc187321905)

[7. Processing of data and statistical analysis 8](#_Toc187321906)

# Patients and clinical-pathological data collection

The study protocol was approved by the Ethics Committee of the Reina Sofia Hospital (COLO-NK, Committee Reference 4884, version 1.0 – 01/12/2020). Patients included in this study (n=25) were enrolled between 2021 and 2024 according to the following inclusion criteria:

1. Age over 18 years or older.
2. Diagnosed with mCRC (stage IV TNM classification).
3. Wild-type RAS status.
4. Scheduled to receive cetuximab (anti-EGFR) as first-line treatment.
5. Signed informed consent to participate in the study.

Peripheral blood samples (±24 ml) were collected in ACD-B tubes (BD Vacutainer, New Jersey, US) from each patient prior to cetuximab treatment. *Non-responders* (NR, n=14) were defined as patients who progressed within 9 months of cetuximab treatment., while those who had progressed (or not) after 9 months were classified as *responders* (R, n=17). This cutoff was selected based on PFS data reported in pivotal randomized trials such as CRYSTAL and FIRE-3, where median PFS among RAS wild-type mCRC patients treated with cetuximab ranged from 8 to 9 months. A 9-months cutoff was thus considered to represent a clinically meaningful duration of disease control and to identify patients who derived sustained benefit from therapy.

The clinical-pathological data evaluated for each patient are summarized in **Appendix 1 (AP1) – Table 1**.

**AP1 – Table 1. Clinical-pathological data of the patients included in the study**.

| Variable | Number of patients (n=31) | Percentage |
| --- | --- | --- |
| Gender  *Male*  *Female* | 24  7 | 77.42 %  22.58 % |
| Age (years) | 64.71$\boldsymbol{\pm}$1.48ª | |
| Tumour size (cm) | 4.31$\boldsymbol{\pm}$0.31ª | |
| Location  *Colon (left)*  *Rectum*  *Other* | 22  8  1 | 70.97 %  25.8 %  3.23 % |
| *WHO* grade  *Low*  *Moderate*  *High*  *N/A^b^* | 6  21  2  2 | 19.35 %  67.75 %  6.45 %  6.45 % |
| Resectability  *Potential*  *Unresectable* | 10  21 | 32.26 %  67.74 % |
| Vascular invasion  *Yes*  *No*  *N/A^b^* | 9  10  12 | 29.03 %  32.26 %  38.71 % |
| Lymphatic invasion  *Yes*  *No*  *N/A^b^* | 11  8  12 | 35.48 %  25.81 %  38.71 % |
| Perineural invasion  *Yes*  *No*  *N/A^b^* | 9  10  12 | 29.03 %  32.26 %  38.71 % |
| ECOG  *0-1*  $\boldsymbol{\geq}$*2* | 28  3 | 90.32 %  6.68 % |
| Surgery primary tumour  *Yes*  *No* | 19  12 | 61.29 %  38.71 % |
| Adjuvant treatment  *Yes*  *No* | 8  23 | 25.80 %  74.20 % |
| Chemotherapy  *5-FU*  *FUOX*  *FOLFOX*  *FUIRI* | 4  8  16  3 | 12.9 %  25.8 %  51.62 %  9.68 % |
| Metastases  *One location*  *More than one location* | 15  16 | 48.39 %  51.61 % |
| Surgery metastases  *Yes*  *No* | 12  19 | 38.71 %  61.29 % |

ª Mean $\pm$SEM; ^b^ N/A: not available

# Immunophenotyping of Peripheral Blood Mononuclear Cells (PBMCs) and NK cell purification

Peripheral blood mononuclear cells (PBMCs) were isolated by Ficoll gradient (LymphoPrep, StemCell-Technologies, Vancouver, Canada) following the manufacturer’s instructions. One sample from R group was discarded due to the poor quality. Immunophenotyping of PBMCs from patients was performed by flow cytometry analysis with FlowJo software version v10.5.3 (FlowJo, Ashland, OR, USA) on LSR-Fortessa flow cytometer (BD Biosciences, New Jersey, US), using the panel of antibodies listed in **AP1 – Table 2.**

**AP1 – Table 2.** Monoclonal antibodies used in flow cytometry analysis for immunophenotyping.

| Monoclonal antibody | Fluorochrome | Commercial provider | Reference |
| --- | --- | --- | --- |
| Fixable Viability Stain (FVS) 510 | BV510 | BD Biosciences | 564406 |
| CD45 | BB700 | BD Biosciences | 746090 |
| CD3 | APC-Cy7 | BD Biosciences | 557832 |
| CD56 | BV421 | BD Biosciences | 562751 |
| CD16 | BV786 | BD Biosciences | 563690 |
| CD14 | PE-Cy5 | Invitrogen Thermo Fisher | #15-0149-42 |
| CD19 | PE-Cy5 | BD Biosciences | 555414 |
| CD25 | PE | BD Biosciences | 557138 |
| CD57 | FITC | BD Biosciences | 555619 |
| CD69 | APC-R700 | BD Biosciences | 565154 |
| NKG2A | BV711 | BD Biosciences | 747919 |
| NKG2C | BV650 | BD Biosciences | 748165 |
| NKp30 | BV605 | BD Biosciences | 563384 |
| NKp44 | APC | Invitrogen Thermo Fisher | #17-3369-42 |
| NKp46 | PE-Cy7 | BD Biosciences | 562101 |
| TIGIT | BUV395 | BD Biosciences | 741182 |

PBMCs were incubated with the antibodies for 20 min at room temperature (RT) in dark, then washed using Phosphate Buffered Saline (PBS)/2% Foetal Bovine Serum (FBS) and fixed with CytoFix Fixation Buffer solution. All antibodies were titrated prior to use and spectral overlap compensation was performed automatically using the BD FACSDiva software with CompBeads Plus (BD Biosciences, New Jersey, US). 8-peaks Rainbow Compensation Particles Set (BD Biosciences, New Jersey, US) were used prior to each experiment for standardization. The gating strategy used for the flow cytometry analysis is shown in **AP1 – Figure 1**. NK cells were typically defined as CD56^Dim^ CD16^+^, with potent cytotoxicity activity, and CD56^+^ (Bright) CD16^-^, known as immunoregulators. However, several studies showed that there is a small population of CD56 Bright which express CD16 at low concentrations, but with important roles in tumour cell death because of they acquire cytotoxic capacity. Moreover, their predominancy is enhanced when NKs are in a cancer and inflammatory environment, promoted by cytokines such as IL-15, or even in other diseases such obesity (Cooper, Fehniger, and Caligiuri 2001; Naujoks et al. 2020; Poznanski et al. 2018; Shin et al. 2023; Takahashi et al. 2007; Wagner et al. 2017). Then, we divided NK subpopulations in three groups: CD56^Bright^ CD16^low/-^ (“**Immunoregulatory NKs**”) CD56^Dim^ CD16+ (“**Cytotoxic NKs**”) and CD56^Dim^ CD16- (**less cytotoxic**). For some analyses we grouped CD56^Bright^ and CD56^Dim^ CD16- together as “**Non-cytotoxic NKs**”.

To generate t-distributed stochastic neighbor embedding (tSNE) plots, NKs from each group were downsampled and concatenated to 53124 cells per response (NR and R groups) using the DownSample V3 plugin, ensuring an equal number of cells across all samples. The same procedure was performed for NKT cells, but downsampling and concatenating to 10285 cells per group. Clusters were generated based on marker expression for each event using the tSNE feature in FlowJo.

Highly purified NK cells were obtained from PBMCs from patients by negative selection using magnetic beads (Miltenyi Biotech, Bergisch Gladbach, Germany) and stored at -80ºC until proteomic analysis. For subsequent co-culture assays, NK cells were isolated from healthy donors and expanded in vitro for 14 days in NK MACS Basal Medium supplemented with NK MACS Supplement (Miltenyi Biotech, Bergisch Gladbach, Germany), 5% human serum (Sigma-Aldrich, San Luis, Misuri, US) and 500 IU/ml IL-2 and 140 IU/ml IL-15 (Miltenyi Biotech, Bergisch Gladbach, Germany).

# Data-independent acquisition (DIA) proteomics analysis.

DIA proteomic analysis was performed in purified NK cells from NR patients (n=10) and R patients (n=9). Proteins were extracted using RIPA buffer lysis (Sigma-Aldrich, San Luis, Missouri, US), following an ultracentrifugation at 10,000 g at 4ºC for 30 minutes, and supernatant was frozen at -80ºC until proteomic studies. After thawing, proteins were precipitated using methanol:chloroform and total protein concentration was measured by Qubit Protein Assay Kit (Thermo Fisher Scientific, Waltham, MA, USA). Proteins were digested using the iST kit (PreOmics, Germany) according to the manufacturer’s instructions. All samples were analyzed by LC-MS/MS using the DIA-PASEF mode on the EvosepOne-TIMSTOF-Flex Clinical Proteomics Platform IMSMI. One NR sample and two R samples failed quality control and were excluded from further analysis. After MS data acquisition, 4914 proteins were identified and quantified with a False Discovery Rate (FDR) of 5%.

Differentially expressed proteins ($\pm$2 *fold-change* and *p-value* > 0.05) between groups were identified using the Metaboanalyst platform (<https://www.metaboanalyst.ca/>), and protein-protein interaction network was performed using STRING (<https://string-db.org/>). Finally, Gene Sets Enrichment Analysis (GSEA) was used for functional enrichment, considering significant pathways when FDR ≤ 0.25. The mass spectrometry proteomics data have been deposited to the *ProteomeXchange Consortium* via the PRIDE partner repository with the dataset identifier PXD057989.


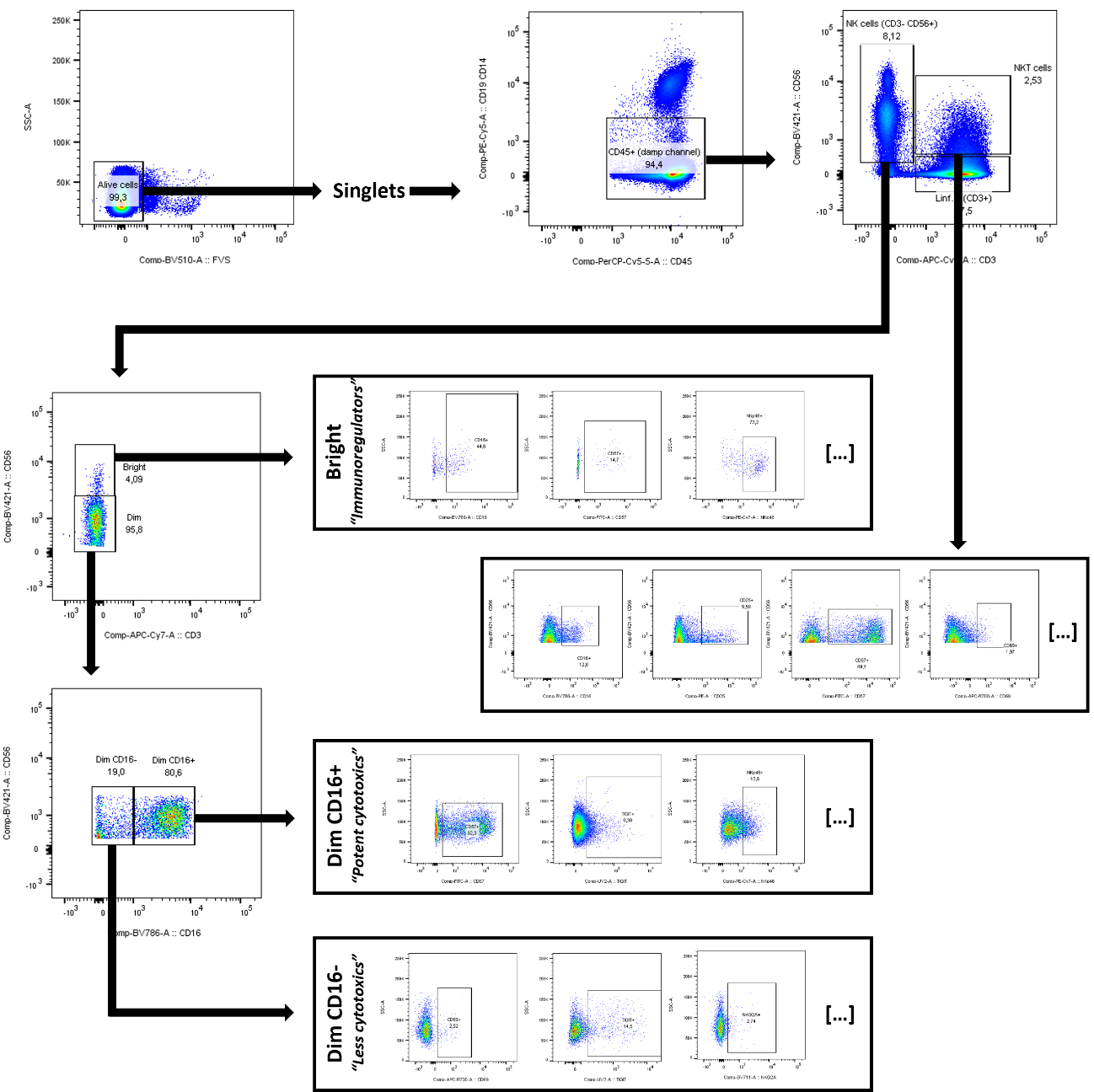


**AP1 – Figure 1.** Gating strategy for immunophenotyping using flow cytometry.

# Human CRC cell lines

SW48 (RAS^wt^) human CRC cell line was kindly provided by Prof. Teresa Roldán (University of Córdoba), while Caco2 (RAS^wt^) and HT29 (RAS^wt^ BRAF^mut^) human CRC cell lines were obtained from American Type Culture Collection (ATCC). All cell lines used were authenticated and mycoplasma-free. SW48 and HT29 cells were maintained in McCoy’s 5A medium (Capricorn Scientific, Ebsdorfergrund, Germany) supplemented with 10% FBS (Biowest, Nuaillé, France) and 1% Zellshield (Minerva Biolabs, Berlin, Germany), and Caco2 were maintained in DMEM (Sigma-Aldrich, San Luis, Misuri, US) supplemented with 20% FBS, 1% ZellShield and 1% MEM non-essential aminoacids (ThermoFisher Scientific, Waltham, Massachusetts, US). All CRC cell lines were incubated in humified incubator at 37ºC / 5 % CO_2_ (*v/v*).

# Generation of three-Dimensional (3D) tumour spheroids for cytotoxicity assays

To form 3D tumour spheroids, 20 000 cells per well were mixed with Matrigel growth factor-reduced basement membrane matrix (Corning, New York, US) at a 1:1 dilution in 50 µl drops. The supplemented media was Advanced DMEM/F12 (Gibco Thermo Fisher Scientific, Waltham, MA, USA), 1% Zellshield, 2% B27 supplement without vitamin A and 1% N2 supplement (Thermo Fisher Scientific, Waltham, MA, USA). Spheroids were refreshed with complete medium and monitored by light microscopy twice a week.

When spheroids were fully formed (usually at day 10), they were digested with TrypLE Express (Gibco Thermo Fisher Scientific, Waltham, MA, USA) for 15 min at 37ºC and labelled with Carboxyfluorescein succinimidyl ester (CFSE) solution (7-AAD/CFSE Cell-Mediated Cytotoxicity Kit; Cayman Chemical Company, Ann Arbor, Michigan, US) during 15 minutes and 37ºC. Then, single cell suspension, representing the target single spheroids, were washed twice with medium with 10% FBS and cocultured with NK cells for 24 hours in 5:1 (effector:target) ratio in presence or absence of 10 µg/ml cetuximab (anti-EGFR, Erbitux, Merck KGaA, Darmstadt, Germany), 25 µg/ml tiragolumab (anti-TIGIT, RG6058, MTIG7192A, Selleck Chemicals, Cologne, Germany) or 20 µl/10^7^ millions of cells of FcR blocking reagent (Miltenyi Biotech, Bergisch Gladbach, Germany) as a control. Finally, each sample were stained with 7-AAD Viability Dye 1X and cell death was analysed by flow cytometry, gating on CFSE+ cells and then 7-AAD stained cells (target cell death, **AP1- Figure 2**).


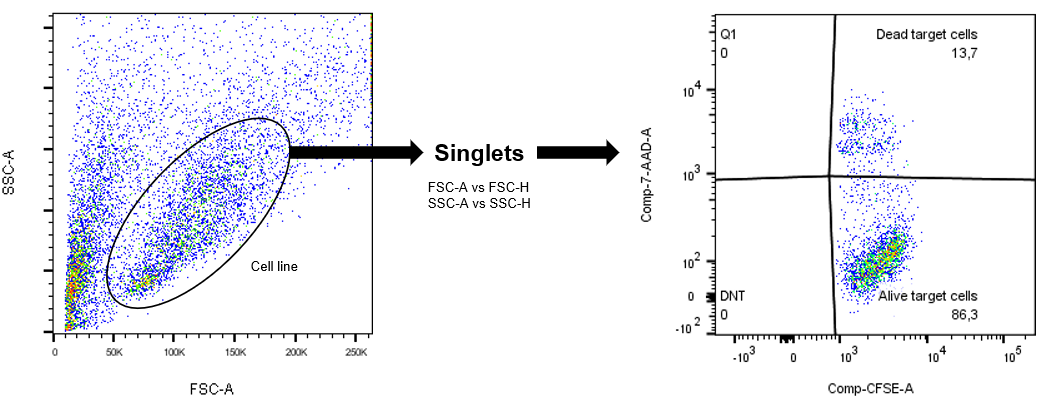


**AP1 – Figure 2.** Gating strategy used in the flow cytometry analysis of cytotoxicity assay.

# Tumour spheroids infiltration assays

For spheroid infiltration assays, 10,000 cells per well were cultured in a 96-well U bottom Nunclon Sphera plate (Thermo Fisher Scientific, Waltham, MA, USA) using RPMI medium supplemented with 1% Zellshield, 1% Glutamax (Gibco Thermo Fisher Scientific, Waltham, MA, USA), 10% FBS and 1% Sodium Piruvate (Gibco Thermo Fisher Scientific, Waltham, MA, USA).

Five days later, the IN/OUT technique was performed as previously described (Courau et al. 2019). At this point, spheroids contained around 30 000 cells and co-cultures were initiated by adding 150 000 freshly isolated NK cells (ratio effector:target 5:1) in complete RPMI media to a final volume of 200 µl. Four wells per condition were seeded. Cetuximab (10 µg/ml) and tiragolumab (25 µg/ml) were added by the same time as NK cells. Twenty-four hours later, the coculture is finished. In brief, medium was removed and the non-infiltrating NK cells (OUT), were washed in FACS buffer and saved for flow cytometry staining. The IN fraction (spheroid with infiltrating NK cells) was washed twice with sterile DPBS (Gibco Thermo Fisher Scientific, Waltham, MA, USA) to ensure that all non-infiltrating and peripheral NK cells were removed and then trypsinized for 5 minutes to obtain a single cell suspension. Both OUT and IN fractions were finally stained for flow cytometry analyses using the antibodies listed in **AP1 – Table 3**. The gating strategy used is described in **AP1 – Figure 3.**

**AP1 – Table 3**. Monoclonal antibodies used in flow cytometry analysis for infiltration assays.

| Monoclonal antibody | Fluorochrome | Commercial provider | Reference |
| --- | --- | --- | --- |
| Annexin V | FITC | Miltenyi Biotech | 130-093-060 |
| EpCAM (CD326) | BV605 | BD Biosciences | 563182 |
| DAPI | DAPI | Thermo Fisher | D3571 |
| CD45 | PE | BD Biosciences | 555483 |
| CD56 | AF700 | BD Biosciences | 557919 |
| CD16 | APC-H7 | BD Biosciences | 560195 |
| CD25 | BV650 | BD Biosciences | 563718 |
| CD69 | PE-Cy5 | BD Biosciences | 555532 |
| NKG2D | PE-Vio770 | Miltenyi Biotech | 130-104-209 |
| CD137 (4-1BB) | APC | BD Biosciences | 55890 |


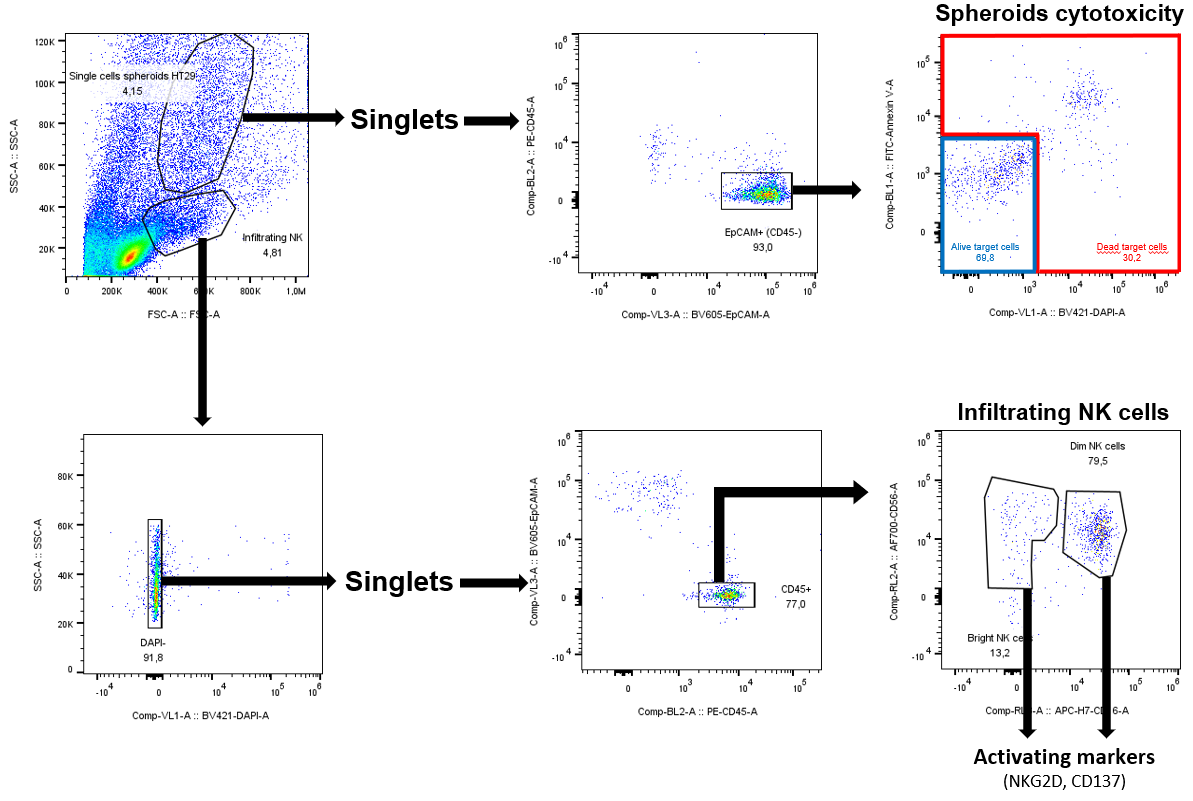


**AP1 – Figure 3.** Gating strategy used in the flow cytometry analysis of infiltration assays.

# Processing of data and statistical analysis

Clinicopathological data were analysed by *Fisher’s exact* and *t-student* test and overall survival (OS) and time to progression (TTP) were examined using *Kaplan-Meier* and *log rank* survival analyses, with *NR group* as reference. The optimal cut-off values were determined based on the median expression values at diagnostic, and Receiver Operating Characteristic (ROC) curves were used to evaluate the diagnostic performance of each marker. *One-way ANOVA* test was performed to compare between groups, *Mann-Whitney* for parametric samples and *Krustal-Wallis* test for non-parametric samples. Statistical significance was set at *p-value* < 0.05 (two-tailed, 95% confidence interval) using GraphPad Prism 9 (software v9.0.0, San Diego, California, US).
