# Supplementary material for: Imbalanced NK cell subpopulations and TIGIT expression limit cetuximab efficacy in colorectal cancer: A promising target for treatment enhancement
Source: Clin Transl Med. 2025 Jun 9;15(6):e70351. doi: 10.1002/ctm2.70351 (PMC12148951; doi:10.1002/ctm2.70351)
Supplement: Supplementary file 2 — Supporting Information [file CTM2-15-e70351-s002.docx]

**Appendix 2: Supplementary tables**

**Supplementary Table 1.** Association of clinical-pathological variables with the response to cetuximab.

| Parameters | Non responders (NR) | Responders (R) | P value  *(Fisher’s exact test)* |
| --- | --- | --- | --- |
| Patient’s characteristics | | | |
| Gender  *Male*  *Female* | 10 (71.43 %)  4 (28.57 %) | 14 (82.35 %)  3 (17.65 %) | 0.6705 |
| Age (*years*$\boldsymbol{)}$ | 67.07$\pm$2.55^a^ | 62.76$\pm$1.63^a^ | 0.1518 |
| Histopathological characteristics | | | |
| Tumour size *(cm)* | 4.27$\pm$0.33^a^ | 4.39$\pm$0.50^a^ | 0.8371 |
| Location  *Colon (left)*  *Rectum*  *Other* | 10 (71.43 %)  3 (21.43 %)  1 (7.14 %) | 12 (70.59 %)  5 (29.41 %)  0 (0 %) | 0.6974 |
| Grade at diagnosis  *G1-2*  *G3-4*  *N/A* | 10 (71.42 %)  2 (14.29 %)  2 (14.29 %) | 17 (100 %)  0 (0 %)  0 (%) | 0.0811 |
| Clinical data | | | |
| Cetuximab cycles received | 9.71$\pm$1.29^a^ | 21.71$\pm$3.13^a^ | ***0.0027*** |
| ECOG  *0-1*  *<2* | 11 (78.57 %)  3 (21.43 %) | 17 (100 %)  0 (0 %) | 0.0810 |
| Surgery primary tumour  *Yes*  *No* | 8 (57.14 %)  6 (42.86 %) | 11 (64.71 %)  6 (35.26 %) | 0.7241 |
| Number of metastases  *One*  *More than one* | 5 (35.71 %)  9 (64.29 %) | 10 (58.82 %)  7 (41.18 %) | 0.2852 |
| Surgery metastases  *Yes*  *No* | 3 (21.43 %)  11 (78.57 %) | 8 (41.06 %)  9 (52.94 %) | 0.2580 |

ª Mean $\pm$SEM

**Supplementary Table 2.** Correlations between clinical-pathological data of *non-responders* (NR) and *responders* (R) with overall survival (OS) and time to progression (TTP).

|  | Non responders (NR) | | | | Responders (R) | | | |
| --- | --- | --- | --- | --- | --- | --- | --- | --- |
| Variables | **OS** | | **TTP** | | **OS** | | **TTP** | |
|  | HR (95% CI) | P- value | HR (95% CI) | P-value | HR (95% CI) | P-value | HR (95% CI) | P-value |
| Age (median)  < *66 years*  $\boldsymbol{\geq}$ *66 years^R^* | 0.936  (0.284-3.088) | 0.9098 | 0.422  (0.095-1.872) | 0.1118 | 1.000  (0.091-11.03) | >0.9999 | 0.295  (0.085-1.022) | 0.0949 |
| Sex  *Male*  *Female^R^* | 2.930  (0.587-14.62) | ***0.0497*** | 1.596  (0.431-5.904) | 0.4037 | Undefined | 0.3659 | 0.788  (0.185-3.358) | 0.7610 |
| Tumour size* (mean)  *< 4.4 cm*  $\boldsymbol{\geq}$ *4.4 cm^R^* | 1.102  (0.353-3.444) | 0.8590 | 0.426  (0.112-1.616) | 0.1069 | 2.320  (0.240-22.44) | 0.4711 | 1.093  (0.316-3.781) | 0.8869 |
| Surgery primary tumour  *Yes^R^*  *No* | 0.856  (0.285-2.571) | 0.7687 | 0.444  (0.132-1.489) | 0.0931 | 0.167  (0.012-2.274) | 0.0669 | 0.415  (0.106-1.618) | 0.1479 |
| Location^#^  *Left colon*  *Rectum^R^* | 3.507  (0.470-26.17) | ***0.0331*** | 2.433  (0.540-10.96) | 0.0860 | Undefined | 0.2207 | 0.249  (0.072-0.859) | ***0.0354*** |
| Number of metastases  *One location^R^*  *More than one* | 1.778  (0.515-6.138) | 0.2589 | 1.494  (0.460-4.856) | 0.4272 | 0.331  (0.033-3.338) | 0.3398 | 0.352  (0.086-1.444) | 0.0667 |

*R: reference group*

**One tumour size not available*

*^#^ Other locations not considered for survival analysis*

*Undefined:* insufficient data for calculating Hazard Ratio (*logrank* method)

**Supplementary Table 3.** List of differentially expressed proteins in *non-responders* (NR) patients.

| Protein  name | log2(FC) | p-value |  |
| --- | --- | --- | --- |
| AHNK2 | -12.293 | 1.01E-02 |  |
| BGH3 | -8.8879 | 4.21E-02 |  |
| GOGA4 | -8.023 | 4.40E-10 |  |
| AGRV1 | -6.4403 | 3.82E-04 |  |
| SUV3 | -6.3304 | 3.25E-08 |  |
| GOLM2 | -6.2188 | 1.37E-04 |  |
| KS6A6 | -6.0866 | 6.56E-05 |  |
| CL16A | -5.9162 | 5.71E-03 |  |
| GBRL1 | -5.7535 | 2.00E-05 |  |
| NU2M | -5.7379 | 1.87E-05 |  |
| ATD3B | -5.7252 | 2.18E-05 |  |
| RPB1 | -5.6105 | 2.64E-09 |  |
| FA83G | -5.5913 | 1.32E-02 |  |
| PHF2 | -5.5355 | 1.36E-03 |  |
| INP5K | -5.5149 | 2.48E-02 |  |
| TM230 | -5.4646 | 8.45E-04 |  |
| BUD13 | -5.3699 | 1.07E-03 |  |
| GSKIP | -5.2334 | 1.04E-03 |  |
| CHCH9 | -5.2139 | 6.56E-06 |  |
| BORC8 | -5.1582 | 9.53E-06 |  |
| ELMD2 | -5.1365 | 1.65E-04 |  |
| LMOD2 | -5.1199 | 1.68E-04 |  |
| MIX23 | -5.113 | 5.27E-06 |  |
| VPS53 | -5.1028 | 3.22E-04 |  |
| RM22 | -5.097 | 2.99E-03 |  |
| TNG2 | -5.0476 | 4.47E-03 |  |
| PRIO | -5.043 | 2.85E-04 |  |
| TBA3C | -5.0229 | 1.05E-03 |  |
| TUT7 | -5.0226 | 1.83E-09 |  |
| SPON2 | -5.0145 | 2.80E-03 |  |
| CLCKB | -4.9923 | 1.95E-03 |  |
| K1C17 | -4.9889 | 1.86E-02 |  |
| LCLT1 | -4.9499 | 2.43E-04 |  |
| RPA43 | -4.9312 | 6.30E-04 |  |
| BOLA1 | -4.918 | 1.98E-03 |  |
| DHSD | -4.8297 | 7.83E-04 |  |
| RFXAP | -4.8162 | 2.85E-03 |  |
| SLBP | -4.7518 | 2.45E-03 |  |
| RIPL1 | -4.7366 | 1.48E-03 |  |
| PLCA | -4.709 | 9.98E-04 |  |
| IGHD | -4.6853 | 1.34E-02 |  |
| LMA2L | -4.656 | 8.23E-04 |  |
| USF2 | -4.5862 | 6.05E-05 |  |
| EMC10 | -4.5778 | 6.36E-04 |  |
| DYH8 | -4.5363 | 1.64E-02 |  |
| AT2C1 | -4.5285 | 1.38E-03 |  |
| BDH | -4.5222 | 2.44E-04 |  |
| T179B | -4.5175 | 1.92E-03 |  |
| K2C4 | -4.5128 | 3.79E-10 |  |
| TM223 | -4.5063 | 5.92E-03 |  |
| MUC18 | -4.4799 | 1.27E-04 |  |
| NDC1 | -4.4585 | 4.35E-03 |  |
| CDK2 | -4.4523 | 3.37E-12 |  |
| MGAT1 | -4.4386 | 4.97E-04 |  |
| AP5M1 | -4.4106 | 4.01E-02 |  |
| PYRD | -4.3884 | 2.54E-05 |  |
| KIAS1 | -4.365 | 7.28E-04 |  |
| COPZ2 | -4.3643 | 5.01E-05 |  |
| DPP8 | -4.3528 | 1.46E-03 |  |
| COXM2 | -4.3323 | 3.82E-05 |  |
| CRCM1 | -4.3114 | 2.04E-04 |  |
| RBFA | -4.2582 | 7.48E-04 |  |
| TM245 | -4.2577 | 1.83E-03 |  |
| 4ET | -4.24 | 3.10E-03 |  |
| PT100 | -4.2398 | 4.46E-04 |  |
| DTX3 | -4.2236 | 1.24E-04 |  |
| STX18 | -4.2199 | 3.14E-03 |  |
| Protein  name | | **log2(FC)** | **p-value** |
| UVRAG | -4.2085 | 5.65E-09 |  |
| CCD50 | -4.202 | 6.04E-04 |  |
| NEMP1 | -4.1971 | 6.30E-04 |  |
| P2RX4 | -4.1802 | 9.70E-05 |  |
| SETMR | -4.1739 | 3.69E-04 |  |
| TIDC1 | -4.1563 | 5.07E-05 |  |
| ZFY | -4.1545 | 1.53E-02 |  |
| MA7D1 | -4.135 | 2.21E-04 |  |
| S35B2 | -4.1302 | 3.55E-03 |  |
| HV551 | -4.0808 | 2.38E-03 |  |
| NDC80 | -4.0676 | 5.64E-05 |  |
| SCAM4 | -4.0672 | 1.34E-04 |  |
| SESN2 | -4.0337 | 7.72E-04 |  |
| TBCD7 | -4.0312 | 3.69E-05 |  |
| DNJB2 | -4.0273 | 7.54E-04 |  |
| SF3B5 | -4.0201 | 5.16E-04 |  |
| TDIF1 | -4.0103 | 3.65E-05 |  |
| CPN2 | -4.0079 | 1.50E-02 |  |
| WDR13 | -4.004 | 1.64E-04 |  |
| ST65G | -3.9872 | 7.03E-04 |  |
| RL1D1 | -3.9743 | 9.56E-05 |  |
| CERS2 | -3.9743 | 5.78E-04 |  |
| PTGR2 | -3.9627 | 1.50E-03 |  |
| CAH1 | -3.9569 | 1.43E-02 |  |
| APLD1 | -3.9417 | 2.49E-03 |  |
| YRDC | -3.9343 | 2.84E-04 |  |
| ARL15 | -3.9246 | 1.13E-03 |  |
| PI3R5 | -3.9199 | 2.20E-03 |  |
| AF17 | -3.9156 | 1.36E-04 |  |
| SRP19 | -3.9142 | 2.98E-04 |  |
| ORC3 | -3.9114 | 8.77E-04 |  |
| MOC2B | -3.9065 | 8.81E-04 |  |
| NCDN | -3.9051 | 1.54E-03 |  |
| LTOR5 | -3.9031 | 4.70E-06 |  |
| AFF4 | -3.8813 | 1.21E-05 |  |
| MMTA2 | -3.8794 | 2.05E-04 |  |
| TIM23 | -3.8734 | 4.87E-03 |  |
| CLP1 | -3.8662 | 7.92E-05 |  |
| RM16 | -3.8657 | 3.07E-03 |  |
| KLF3 | -3.8352 | 3.46E-04 |  |
| NB5R4 | -3.8321 | 3.44E-02 |  |
| MICU1 | -3.8237 | 4.86E-04 |  |
| AMBP | -3.8148 | 3.97E-02 |  |
| CD81 | -3.8135 | 2.47E-04 |  |
| PP1RA | -3.8077 | 6.90E-05 |  |
| KITM | -3.8066 | 1.16E-03 |  |
| PECR | -3.795 | 2.21E-03 |  |
| LTV1 | -3.7872 | 4.52E-04 |  |
| MGME1 | -3.7788 | 7.12E-03 |  |
| RN220 | -3.771 | 7.22E-05 |  |
| TMX2 | -3.767 | 3.21E-04 |  |
| PLPL1 | -3.7583 | 2.40E-05 |  |
| SNX8 | -3.7555 | 6.95E-05 |  |
| NOL9 | -3.7411 | 3.28E-03 |  |
| PRAG1 | -3.7389 | 2.56E-05 |  |
| MARF1 | -3.7296 | 2.59E-05 |  |
| SMG9 | -3.7266 | 3.21E-03 |  |
| ACBD6 | -3.7203 | 2.38E-03 |  |
| NKAP | -3.6904 | 4.35E-05 |  |
| AKA11 | -3.6873 | 1.03E-04 |  |
| IPRI | -3.6836 | 5.00E-06 |  |
| WDR12 | -3.6736 | 1.22E-06 |  |
| DHCR7 | -3.6645 | 5.35E-05 |  |
| LGAT1 | -3.6605 | 3.65E-03 |  |
| DDB2 | -3.6557 | 2.34E-05 |  |
| UBR5 | -3.6506 | 6.57E-06 |  |
| VPS51 | -3.6429 | 5.35E-06 |  |
| Protein  name | **log2(FC)** | **p-value** |  |
| ATP8 | -3.6361 | 1.56E-04 |  |
| TM128 | -3.6321 | 1.04E-04 |  |
| CCNL2 | -3.6317 | 1.24E-05 |  |
| FA98C | -3.6256 | 5.41E-03 |  |
| DJC21 | -3.6234 | 1.79E-03 |  |
| DYN3 | -3.6231 | 1.23E-05 |  |
| ASURF | -3.6191 | 4.61E-05 |  |
| TRM1 | -3.6181 | 2.97E-04 |  |
| RM42 | -3.6149 | 5.36E-04 |  |
| CTND1 | -3.6142 | 6.49E-03 |  |
| MED16 | -3.6105 | 2.15E-04 |  |
| NSE4A | -3.6098 | 4.75E-05 |  |
| CA198 | -3.6025 | 1.72E-04 |  |
| THG1 | -3.5885 | 1.04E-04 |  |
| FABD | -3.5873 | 3.90E-05 |  |
| F184A | -3.5854 | 2.96E-04 |  |
| P3H1 | -3.585 | 1.46E-04 |  |
| L2GL1 | -3.5756 | 3.72E-05 |  |
| UXT | -3.5668 | 1.24E-05 |  |
| KDM4B | -3.5664 | 3.39E-05 |  |
| CREL2 | -3.5634 | 1.64E-04 |  |
| DUS11 | -3.5624 | 1.74E-03 |  |
| AGRE2 | -3.5547 | 4.13E-03 |  |
| NMRL1 | -3.5465 | 3.78E-06 |  |
| NOB1 | -3.5461 | 5.99E-04 |  |
| NDK6 | -3.5459 | 6.48E-04 |  |
| TRAF2 | -3.5395 | 2.91E-05 |  |
| NAA35 | -3.5312 | 1.29E-05 |  |
| INT11 | -3.5284 | 2.90E-03 |  |
| SAMD1 | -3.5264 | 6.11E-07 |  |
| EXOC2 | -3.5231 | 1.59E-05 |  |
| M3K4 | -3.5226 | 3.11E-05 |  |
| KS6B1 | -3.5216 | 9.21E-05 |  |
| TRIM4 | -3.5183 | 2.81E-04 |  |
| NUD4B | -3.5162 | 1.78E-06 |  |
| UBP11 | -3.5119 | 6.71E-10 |  |
| POTEI | -3.5118 | 2.41E-03 |  |
| RN114 | -3.5089 | 1.81E-07 |  |
| MED1 | -3.5011 | 2.62E-05 |  |
| RPA1 | -3.4977 | 1.38E-04 |  |
| TRM2A | -3.4956 | 3.13E-05 |  |
| NDUF7 | -3.494 | 1.91E-04 |  |
| TXD15 | -3.4933 | 1.52E-04 |  |
| P5CR2 | -3.4902 | 5.23E-05 |  |
| MOG1 | -3.4879 | 5.39E-05 |  |
| CC168 | -3.4847 | 3.69E-04 |  |
| LSG1 | -3.4835 | 5.46E-06 |  |
| PCDGD | -3.4796 | 2.04E-04 |  |
| PPCT | -3.4763 | 8.81E-05 |  |
| SAP30 | -3.4681 | 1.84E-04 |  |
| F210A | -3.4658 | 4.17E-05 |  |
| CR025 | -3.4605 | 5.13E-07 |  |
| XPF | -3.4599 | 1.10E-05 |  |
| GDF2 | -3.4583 | 1.19E-02 |  |
| UBP16 | -3.4572 | 1.48E-04 |  |
| PCMD1 | -3.4508 | 1.60E-05 |  |
| MED8 | -3.45 | 7.05E-06 |  |
| NCTR1 | -3.4423 | 3.28E-04 |  |
| PEAK3 | -3.4355 | 1.77E-04 |  |
| OSBL2 | -3.4289 | 1.16E-04 |  |
| COX20 | -3.4285 | 5.20E-06 |  |
| PALLD | -3.4189 | 5.94E-05 |  |
| RM02 | -3.4136 | 4.66E-06 |  |
| TMM65 | -3.4104 | 1.95E-04 |  |
| COQ8B | -3.4042 | 8.86E-05 |  |
| VMAC | -3.4031 | 5.57E-04 |  |
| MI4GD | -3.4027 | 4.81E-06 |  |
| Protein  name | **log2(FC)** | **p-value** |  |
| TRIM5 | -3.3962 | 2.65E-05 |  |
| KI21A | -3.3941 | 7.87E-04 |  |
| ENAH | -3.391 | 2.15E-06 |  |
| NUBP2 | -3.3899 | 1.64E-04 |  |
| KAIN | -3.3864 | 1.46E-03 |  |
| BCAS3 | -3.3858 | 6.86E-06 |  |
| SPTN4 | -3.3641 | 5.07E-05 |  |
| RBL2 | -3.3609 | 4.65E-07 |  |
| UBAP2 | -3.3603 | 2.60E-05 |  |
| GFOD1 | -3.3571 | 1.14E-03 |  |
| SYPM | -3.3559 | 1.22E-05 |  |
| SNPC1 | -3.3446 | 2.22E-05 |  |
| MTDC | -3.3308 | 2.93E-07 |  |
| IQEC1 | -3.3281 | 9.90E-06 |  |
| RN5A | -3.327 | 1.79E-06 |  |
| ACH10 | -3.3195 | 2.20E-06 |  |
| MPRIP | -3.316 | 2.00E-06 |  |
| RASF4 | -3.3149 | 4.54E-04 |  |
| LEG9 | -3.3104 | 9.95E-04 |  |
| RBM28 | -3.3103 | 5.33E-06 |  |
| CRTC3 | -3.3099 | 8.33E-07 |  |
| MRES1 | -3.3018 | 8.10E-05 |  |
| Z3H7B | -3.3013 | 1.84E-06 |  |
| MA1A1 | -3.3007 | 2.65E-06 |  |
| TTC27 | -3.2933 | 5.47E-05 |  |
| VRK2 | -3.2907 | 2.89E-04 |  |
| WWP2 | -3.2875 | 2.79E-04 |  |
| TM41B | -3.28 | 4.03E-06 |  |
| RARR2 | -3.2791 | 8.91E-06 |  |
| GNPTA | -3.2757 | 1.44E-03 |  |
| TM256 | -3.2737 | 3.08E-06 |  |
| PORCN | -3.2735 | 1.97E-04 |  |
| COX1 | -3.2691 | 8.00E-06 |  |
| SE1L1 | -3.2646 | 1.49E-06 |  |
| TATD3 | -3.2591 | 2.71E-06 |  |
| BCS1 | -3.2588 | 2.18E-06 |  |
| IN80E | -3.2576 | 1.35E-06 |  |
| AP4S1 | -3.2568 | 7.61E-06 |  |
| USE1 | -3.2557 | 2.77E-05 |  |
| TAM41 | -3.2556 | 9.26E-07 |  |
| NCOA1 | -3.2536 | 5.13E-05 |  |
| SMG8 | -3.2519 | 2.71E-07 |  |
| CHIO | -3.2442 | 4.13E-05 |  |
| CYTS | -3.2411 | 1.35E-04 |  |
| AP5Z1 | -3.2387 | 3.05E-05 |  |
| RM18 | -3.2386 | 1.14E-07 |  |
| RT21 | -3.2351 | 1.33E-04 |  |
| CTU2 | -3.2275 | 3.19E-07 |  |
| CCD92 | -3.227 | 1.91E-05 |  |
| CALL5 | -3.227 | 2.58E-03 |  |
| PURA1 | -3.2258 | 2.62E-03 |  |
| TPC6B | -3.2138 | 5.71E-10 |  |
| SYNJ2 | -3.2121 | 1.00E-04 |  |
| FBX22 | -3.2109 | 2.56E-06 |  |
| TM160 | -3.2074 | 3.18E-05 |  |
| SO4C1 | -3.1968 | 1.97E-06 |  |
| MSL1 | -3.1966 | 4.08E-06 |  |
| FXL18 | -3.1962 | 9.21E-07 |  |
| TSR1 | -3.1926 | 2.61E-05 |  |
| RM27 | -3.1902 | 2.02E-06 |  |
| IMP3 | -3.1875 | 1.65E-06 |  |
| PBX2 | -3.1872 | 1.94E-07 |  |
| TISD | -3.1865 | 5.72E-05 |  |
| DDX20 | -3.1842 | 2.40E-06 |  |
| ACACB | -3.177 | 3.01E-07 |  |
| PLXD1 | -3.1762 | 6.88E-05 |  |
| MEN1 | -3.1669 | 2.72E-06 |  |
| PHC2 | -3.1659 | 9.46E-06 |  |
| PRP18 | -3.163 | 1.51E-06 |  |
| Protein  name | **log2(FC)** | **p-value** |  |
| DNAS1 | -3.1623 | 5.27E-09 |  |
| IF3M | -3.162 | 9.18E-06 |  |
| UB2G2 | -3.1551 | 1.89E-06 |  |
| RFX5 | -3.1546 | 1.14E-05 |  |
| GPBP1 | -3.1529 | 8.83E-06 |  |
| CS025 | -3.1517 | 1.35E-04 |  |
| DI3L2 | -3.1429 | 4.45E-05 |  |
| NUP42 | -3.1415 | 2.98E-04 |  |
| BAG2 | -3.1403 | 3.02E-05 |  |
| HPS6 | -3.1394 | 2.02E-07 |  |
| TTC5 | -3.1383 | 3.25E-08 |  |
| UIMC1 | -3.137 | 9.62E-06 |  |
| TAF3 | -3.1323 | 7.82E-05 |  |
| WDR48 | -3.1303 | 2.36E-07 |  |
| GNL1 | -3.1233 | 6.98E-09 |  |
| NFX1 | -3.1078 | 1.40E-06 |  |
| HACD4 | -3.1073 | 6.56E-06 |  |
| RM04 | -3.1027 | 8.33E-08 |  |
| RHBD2 | -3.1023 | 2.17E-07 |  |
| NQO1 | -3.1012 | 1.49E-04 |  |
| NSUN5 | -3.1003 | 1.78E-06 |  |
| HIP1R | -3.0943 | 1.04E-05 |  |
| RHG12 | -3.0905 | 2.84E-05 |  |
| RPP29 | -3.0857 | 3.94E-05 |  |
| YPEL5 | -3.0856 | 3.23E-08 |  |
| TDR15 | -3.0841 | 7.79E-08 |  |
| CR063 | -3.0819 | 4.26E-06 |  |
| KPCT | -3.0778 | 4.59E-05 |  |
| KC1G1 | -3.0713 | 1.84E-04 |  |
| TGBR3 | -3.0636 | 1.16E-05 |  |
| COQ8A | -3.0635 | 4.64E-08 |  |
| ZN512 | -3.0581 | 1.85E-06 |  |
| TKTL2 | -3.0567 | 1.65E-07 |  |
| SATT | -3.0563 | 6.60E-04 |  |
| SNIP1 | -3.051 | 1.34E-04 |  |
| MERL | -3.0486 | 1.80E-06 |  |
| CC186 | -3.0465 | 6.35E-06 |  |
| APTX | -3.0463 | 3.56E-07 |  |
| EXOS1 | -3.044 | 5.37E-07 |  |
| WDR90 | -3.0437 | 2.99E-08 |  |
| GON4L | -3.0426 | 3.20E-04 |  |
| P52K | -3.0425 | 5.64E-04 |  |
| TTC19 | -3.0423 | 9.29E-07 |  |
| ARC1A | -3.0399 | 9.16E-08 |  |
| PAXI1 | -3.0377 | 1.49E-05 |  |
| VRK3 | -3.0298 | 1.84E-07 |  |
| FNTB | -3.0296 | 3.22E-06 |  |
| ARAP2 | -3.0289 | 3.09E-07 |  |
| NSL1 | -3.0277 | 1.06E-05 |  |
| AKP8L | -3.0262 | 2.97E-07 |  |
| MCE1 | -3.019 | 2.05E-09 |  |
| SYTL3 | -3.0188 | 5.85E-05 |  |
| ZY11B | -3.005 | 3.22E-04 |  |
| PZRN3 | -3.0009 | 9.54E-08 |  |
| DDR1 | -2.9985 | 1.66E-06 |  |
| UBE2A | -2.9979 | 2.20E-05 |  |
| COQ7 | -2.9904 | 3.79E-06 |  |
| ECSIT | -2.9808 | 1.22E-05 |  |
| ZN740 | -2.9792 | 2.35E-04 |  |
| AKAP9 | -2.9786 | 2.40E-08 |  |
| MTUS1 | -2.975 | 3.53E-07 |  |
| IRF3 | -2.9745 | 5.04E-06 |  |
| AN13D | -2.9689 | 9.20E-06 |  |
| UQCC1 | -2.967 | 2.46E-05 |  |
| CPNE9 | -2.9618 | 4.06E-08 |  |
| UBN1 | -2.9599 | 1.33E-02 |  |
| KRT34 | -2.9509 | 1.00E-07 |  |
| MYOM2 | -2.9484 | 7.90E-08 |  |
| AASD1 | -2.9468 | 2.49E-07 |  |
| Protein  name | **log2(FC)** | **p-value** |  |
| A16L1 | -2.9463 | 5.28E-09 |  |
| PX11B | -2.9448 | 9.28E-05 |  |
| SPS2 | -2.9431 | 1.79E-06 |  |
| ABLM1 | -2.9401 | 2.36E-09 |  |
| GLCI1 | -2.9339 | 2.14E-04 |  |
| DCTN5 | -2.9306 | 3.38E-08 |  |
| MOT9 | -2.9279 | 1.01E-04 |  |
| XPC | -2.9262 | 1.06E-08 |  |
| T184C | -2.9259 | 1.07E-06 |  |
| FCRL6 | -2.9221 | 6.98E-09 |  |
| DCA11 | -2.9219 | 6.81E-08 |  |
| CTSL2 | -2.9178 | 9.22E-08 |  |
| WDR89 | -2.9166 | 8.73E-08 |  |
| DNJC1 | -2.9136 | 2.12E-07 |  |
| PAPS2 | -2.9119 | 2.77E-08 |  |
| SOS1 | -2.9101 | 1.28E-07 |  |
| NAA40 | -2.9084 | 6.48E-08 |  |
| ZC3HD | -2.9084 | 5.03E-07 |  |
| UBX2B | -2.9083 | 2.17E-04 |  |
| MSL2 | -2.9047 | 1.04E-06 |  |
| RTN1 | -2.9044 | 1.41E-05 |  |
| PAR12 | -2.9025 | 8.97E-07 |  |
| FAKD4 | -2.9017 | 1.82E-09 |  |
| MIC25 | -2.9007 | 2.39E-07 |  |
| DNPH1 | -2.8996 | 1.55E-07 |  |
| GPN1 | -2.8926 | 9.20E-08 |  |
| PGFRB | -2.8921 | 6.14E-05 |  |
| INT8 | -2.8882 | 7.22E-08 |  |
| FCSD1 | -2.888 | 5.93E-06 |  |
| ZER1 | -2.8871 | 1.70E-04 |  |
| RIMC1 | -2.8852 | 2.76E-06 |  |
| RICTR | -2.8777 | 3.80E-07 |  |
| DDX59 | -2.8757 | 3.43E-08 |  |
| DGCR8 | -2.873 | 1.10E-07 |  |
| MK10 | -2.8716 | 3.95E-09 |  |
| MS3L1 | -2.8625 | 6.69E-07 |  |
| OTU6B | -2.8624 | 1.75E-09 |  |
| HSC20 | -2.8522 | 4.60E-11 |  |
| SLU7 | -2.8505 | 9.39E-09 |  |
| CO2 | -2.8495 | 1.90E-08 |  |
| SUN1 | -2.8478 | 1.84E-08 |  |
| PIGT | -2.8426 | 5.94E-05 |  |
| TOR1B | -2.8423 | 6.78E-08 |  |
| CDK3 | -2.839 | 5.94E-07 |  |
| DNJA4 | -2.8388 | 1.45E-08 |  |
| NGLY1 | -2.8368 | 1.26E-06 |  |
| GOPC | -2.8355 | 2.10E-05 |  |
| SPG7 | -2.8224 | 1.69E-06 |  |
| NCOR2 | -2.8205 | 6.86E-11 |  |
| P3C2G | -2.8204 | 3.11E-07 |  |
| PLCD1 | -2.8098 | 2.91E-07 |  |
| PYGO2 | -2.8053 | 1.64E-08 |  |
| RBM22 | -2.7991 | 2.20E-08 |  |
| ISCU | -2.7958 | 4.28E-06 |  |
| CHSTC | -2.7861 | 2.68E-04 |  |
| MZT2B | -2.786 | 1.06E-06 |  |
| UBP19 | -2.7859 | 8.40E-09 |  |
| GGT2 | -2.7833 | 6.73E-07 |  |
| UT14A | -2.783 | 4.31E-07 |  |
| GRL1A | -2.7822 | 1.28E-08 |  |
| C99L2 | -2.7807 | 6.93E-04 |  |
| COG4 | -2.7796 | 9.42E-08 |  |
| MED17 | -2.7788 | 6.49E-08 |  |
| RN169 | -2.7758 | 3.32E-06 |  |
| XIRP1 | -2.7625 | 7.58E-09 |  |
| GOGB1 | -2.7606 | 3.57E-09 |  |
| CCNT1 | -2.7593 | 5.26E-08 |  |
| ASF1A | -2.7553 | 8.75E-09 |  |
| RMP | -2.7455 | 5.94E-09 |  |
| Protein  name | **log2(FC)** | **p-value** |  |
| N4BP1 | -2.7345 | 5.63E-10 |  |
| PK3CG | -2.7344 | 2.39E-11 |  |
| RBBP9 | -2.734 | 7.53E-09 |  |
| RN214 | -2.7315 | 3.86E-07 |  |
| CEP43 | -2.7311 | 7.35E-08 |  |
| ALG8 | -2.729 | 3.80E-07 |  |
| DVL2 | -2.7207 | 1.39E-07 |  |
| PPIL2 | -2.7103 | 2.31E-09 |  |
| PDIP2 | -2.7066 | 1.74E-09 |  |
| NIPBL | -2.7041 | 7.52E-12 |  |
| MAEA | -2.7023 | 2.90E-08 |  |
| THUM3 | -2.7005 | 1.64E-09 |  |
| ANS1B | -2.6935 | 1.72E-10 |  |
| GLPK | -2.6898 | 9.23E-07 |  |
| STIM2 | -2.6887 | 2.07E-09 |  |
| RT10 | -2.6857 | 5.88E-07 |  |
| NVL | -2.6836 | 5.85E-09 |  |
| SMAL1 | -2.6817 | 1.55E-10 |  |
| CC90B | -2.6788 | 2.50E-09 |  |
| ARI4B | -2.6753 | 3.83E-08 |  |
| RPR1A | -2.6638 | 1.32E-07 |  |
| KDM5A | -2.6631 | 5.00E-09 |  |
| KVD33 | -2.6521 | 9.90E-05 |  |
| LUZP1 | -2.6514 | 3.51E-11 |  |
| KLC4 | -2.6447 | 5.01E-13 |  |
| T11L1 | -2.6365 | 1.79E-08 |  |
| GID8 | -2.6347 | 1.86E-05 |  |
| UBAP1 | -2.6248 | 4.49E-10 |  |
| DGKQ | -2.6198 | 9.26E-10 |  |
| CHKB | -2.6179 | 3.46E-08 |  |
| MITOK | -2.6126 | 2.01E-03 |  |
| NU188 | -2.6082 | 7.58E-12 |  |
| AAMP | -2.5824 | 2.47E-08 |  |
| ULK3 | -2.5801 | 6.27E-08 |  |
| NEK1 | -2.5693 | 2.52E-12 |  |
| HMCS1 | -2.5653 | 9.41E-13 |  |
| SDS3 | -2.5612 | 8.34E-10 |  |
| OSMR | -2.561 | 1.11E-04 |  |
| ZXDA | -2.5608 | 6.18E-05 |  |
| DMAC1 | -2.5568 | 2.00E-12 |  |
| ARI1B | -2.556 | 5.12E-09 |  |
| RRP1B | -2.5539 | 1.06E-04 |  |
| MDC1 | -2.5531 | 2.65E-09 |  |
| SEPT3 | -2.5517 | 3.39E-11 |  |
| TRNK1 | -2.5491 | 4.59E-10 |  |
| ST17A | -2.5488 | 4.24E-10 |  |
| MYCPP | -2.5461 | 9.07E-13 |  |
| TF2H1 | -2.5403 | 5.28E-05 |  |
| MDEAS | -2.5357 | 2.02E-11 |  |
| NSRP1 | -2.5346 | 7.36E-05 |  |
| IRF6 | -2.5068 | 3.15E-05 |  |
| IMA1 | -2.4898 | 1.64E-12 |  |
| ISOC2 | -2.4717 | 7.06E-12 |  |
| REN3B | -2.471 | 1.53E-03 |  |
| MIO | -2.4493 | 2.50E-09 |  |
| PTBP2 | -2.4401 | 1.03E-13 |  |
| MBLC2 | -2.4362 | 5.85E-16 |  |
| BRPF1 | -2.3808 | 4.61E-05 |  |
| RWDD4 | -2.3646 | 1.82E-03 |  |
| ODO1 | -2.3603 | 1.07E-09 |  |
| IRAK4 | -2.2947 | 2.94E-09 |  |
| ABRAL | -2.2397 | 1.61E-03 |  |
| SMRC2 | -2.2292 | 1.99E-10 |  |
| PDS5B | -2.2245 | 7.66E-11 |  |
| GLMN | -2.2175 | 2.10E-02 |  |
| HTSF1 | -2.2078 | 4.83E-10 |  |
| MFF | -2.1914 | 2.25E-08 |  |
| HEM3 | -2.1878 | 2.15E-04 |  |
| GOGA2 | -2.1708 | 1.44E-12 |  |
| Protein  name | **log2(FC)** | **p-value** |  |
| GFRP | -2.1687 | 9.29E-03 |  |
| MPU1 | -2.1103 | 2.27E-03 |  |
| RAP2C | -2.1095 | 1.02E-03 |  |
| TFP11 | -2.0985 | 4.37E-06 |  |
| CYTSA | -2.0824 | 1.82E-05 |  |
| PGPI | -2.0611 | 2.41E-05 |  |
| SYFM | -1.9945 | 1.76E-05 |  |
| LTN1 | -1.9134 | 7.63E-04 |  |
| LAGE3 | -1.899 | 1.17E-04 |  |
| COX2 | -1.8975 | 1.18E-09 |  |
| GNA1 | -1.8851 | 2.05E-05 |  |
| SSNA1 | -1.8619 | 1.18E-05 |  |
| RER1 | -1.8466 | 6.44E-06 |  |
| DOCK5 | -1.8239 | 2.11E-04 |  |
| LIMD1 | -1.8033 | 3.77E-08 |  |
| SYTL2 | -1.7736 | 8.89E-04 |  |
| LAS1L | -1.7628 | 1.97E-05 |  |
| ARF6 | -1.7271 | 2.65E-05 |  |
| PGTB2 | -1.7103 | 3.06E-05 |  |
| MED18 | -1.6917 | 8.51E-04 |  |
| D39U1 | -1.6797 | 3.89E-03 |  |
| F1142 | -1.6708 | 8.30E-10 |  |
| TCF25 | -1.6393 | 2.58E-04 |  |
| MYL1 | -1.6353 | 2.59E-04 |  |
| NCOR1 | -1.6319 | 1.97E-12 |  |
| TLS1 | -1.6273 | 6.01E-04 |  |
| AR2BP | -1.6252 | 4.12E-05 |  |
| MMAB | -1.6168 | 6.64E-04 |  |
| NCKP1 | -1.6076 | 7.15E-03 |  |
| L10K | -1.5939 | 2.46E-07 |  |
| IDS | -1.5741 | 8.30E-04 |  |
| EXOG | -1.5594 | 2.14E-02 |  |
| SPAT5 | -1.553 | 6.83E-07 |  |
| AAGAB | -1.481 | 9.69E-06 |  |
| INT5 | -1.4634 | 9.01E-06 |  |
| PLPL8 | -1.4596 | 4.23E-02 |  |
| CCD93 | -1.4381 | 7.88E-08 |  |
| FHI2A | -1.431 | 2.07E-05 |  |
| PSME3 | -1.4222 | 1.05E-07 |  |
| KCAB2 | -1.4197 | 1.46E-03 |  |
| DD19B | -1.4104 | 2.89E-04 |  |
| SMAP | -1.3892 | 3.61E-03 |  |
| CPSF3 | -1.373 | 3.19E-05 |  |
| TSN14 | -1.3706 | 1.02E-03 |  |
| LEO1 | -1.3671 | 7.55E-05 |  |
| TM9S1 | -1.3349 | 1.40E-02 |  |
| DDX21 | -1.3323 | 3.77E-04 |  |
| GMPPA | -1.3248 | 1.73E-07 |  |
| SNW1 | -1.3221 | 2.73E-05 |  |
| H4 | -1.3175 | 3.72E-02 |  |
| SGF29 | -1.3106 | 8.10E-04 |  |
| MIRO2 | -1.3066 | 3.26E-06 |  |
| KDM2A | -1.3064 | 9.76E-04 |  |
| KLRF1 | -1.296 | 1.14E-02 |  |
| AFAM | -1.296 | 4.03E-02 |  |
| JADE2 | -1.2928 | 2.83E-03 |  |
| HSP7E | -1.265 | 4.04E-06 |  |
| CPSF2 | -1.2581 | 3.03E-07 |  |
| CYBP | -1.2522 | 4.02E-07 |  |
| NPC1 | -1.232 | 3.41E-04 |  |
| FAM3C | -1.2312 | 1.44E-03 |  |
| CASP7 | -1.2251 | 1.18E-02 |  |
| FTO | -1.2191 | 1.62E-06 |  |
| RPB2 | -1.2189 | 8.28E-06 |  |
| GRK6 | -1.2169 | 1.33E-04 |  |
| DIDO1 | -1.2099 | 2.05E-04 |  |
| GPCP1 | -1.2014 | 1.47E-04 |  |
| NELFE | -1.1983 | 4.25E-06 |  |
| SGPL1 | -1.1907 | 5.05E-05 |  |
| Protein  name | **log2(FC)** | **p-value** |  |
| CNPY4 | -1.1826 | 8.18E-03 |  |
| ARL4C | -1.1805 | 2.40E-02 |  |
| NDUF4 | -1.1695 | 2.58E-05 |  |
| KLDC4 | -1.1646 | 2.23E-04 |  |
| APAF | -1.1589 | 3.90E-03 |  |
| RPB11 | -1.1588 | 1.17E-03 |  |
| NRM | -1.148 | 2.40E-03 |  |
| ZN638 | -1.1351 | 2.33E-04 |  |
| SMU1 | -1.1314 | 4.94E-07 |  |
| CX3C1 | -1.1256 | 3.29E-04 |  |
| RIPK1 | -1.1197 | 3.07E-05 |  |
| DHX36 | -1.1164 | 1.67E-05 |  |
| IKIP | -1.1121 | 4.41E-02 |  |
| EXOS6 | -1.1066 | 4.40E-05 |  |
| AGRG5 | -1.1021 | 1.70E-02 |  |
| ARP19 | -1.0991 | 5.00E-04 |  |
| HYOU1 | -1.0988 | 6.83E-09 |  |
| EPB41 | -1.0977 | 1.56E-07 |  |
| FIBP | -1.0909 | 3.66E-03 |  |
| SC11C | -1.0868 | 5.06E-03 |  |
| BRE1B | -1.0859 | 2.07E-07 |  |
| CNPY3 | -1.0818 | 2.45E-03 |  |
| RFX1 | -1.0809 | 1.21E-03 |  |
| RL6 | -1.0808 | 9.64E-04 |  |
| PTER | -1.0706 | 3.02E-04 |  |
| C42S2 | -1.0681 | 1.13E-02 |  |
| ARI1 | -1.0674 | 1.68E-03 |  |
| PACS1 | -1.0668 | 6.17E-04 |  |
| RAB8A | -1.0526 | 2.82E-04 |  |
| F162A | -1.0404 | 3.74E-04 |  |
| PHF10 | -1.039 | 6.47E-03 |  |
| ELP6 | -1.0385 | 1.29E-02 |  |
| DCMC | -1.0333 | 2.61E-04 |  |
| ROAA | -1.0325 | 1.45E-05 |  |
| ANM2 | -1.0238 | 1.03E-03 |  |
| SDE2 | -1.0205 | 2.08E-03 |  |
| NFAC3 | -1.0179 | 6.55E-03 |  |
| GOGA5 | -1.0164 | 5.40E-03 |  |
| VPS39 | -1.0103 | 2.57E-06 |  |
| RS10 | 1.0006 | 3.91E-06 |  |
| SF3A1 | 1.0021 | 7.85E-07 |  |
| NUDC2 | 1.0046 | 2.98E-03 |  |
| PGM1 | 1.0058 | 1.24E-04 |  |
| VPP3 | 1.0189 | 3.41E-02 |  |
| G45IP | 1.0206 | 3.79E-02 |  |
| ACTA | 1.0237 | 3.88E-04 |  |
| CCDC6 | 1.0263 | 5.95E-03 |  |
| DFFA | 1.0291 | 9.52E-06 |  |
| GET4 | 1.0317 | 9.64E-04 |  |
| EIF3B | 1.0356 | 4.54E-08 |  |
| SLAI2 | 1.036 | 5.29E-03 |  |
| GIPC1 | 1.0419 | 4.64E-02 |  |
| PHAX | 1.0422 | 2.64E-02 |  |
| ACTBL | 1.0434 | 1.12E-03 |  |
| GBF1 | 1.0465 | 6.53E-05 |  |
| CSN7A | 1.0489 | 4.65E-03 |  |
| NPS3A | 1.0504 | 3.65E-03 |  |
| IF140 | 1.0518 | 3.34E-02 |  |
| LNP | 1.0522 | 3.80E-02 |  |
| CTDS1 | 1.0549 | 4.01E-03 |  |
| FAAA | 1.0577 | 3.32E-02 |  |
| PSB10 | 1.0599 | 2.06E-05 |  |
| MK03 | 1.0648 | 1.03E-05 |  |
| RM23 | 1.065 | 3.41E-03 |  |
| CDK7 | 1.069 | 2.42E-02 |  |
| CDN1B | 1.0699 | 1.69E-02 |  |
| HDHD3 | 1.0783 | 4.76E-03 |  |
| SPCS3 | 1.0824 | 1.06E-06 |  |
| PARN | 1.0831 | 2.14E-04 |  |
| Protein  name | **log2(FC)** | **p-value** |  |
| E2AK2 | 1.0868 | 3.45E-06 |  |
| RT28 | 1.0905 | 5.03E-03 |  |
| SYSC | 1.0922 | 9.02E-11 |  |
| EGLN1 | 1.0941 | 4.10E-02 |  |
| TWF1 | 1.0957 | 3.01E-05 |  |
| BUD31 | 1.1014 | 1.01E-03 |  |
| WDR4 | 1.103 | 2.99E-02 |  |
| SSRG | 1.1045 | 2.00E-03 |  |
| IF4G3 | 1.1049 | 7.99E-03 |  |
| MCM2 | 1.1058 | 2.61E-03 |  |
| FES | 1.1172 | 6.42E-03 |  |
| GSK3B | 1.1212 | 4.01E-06 |  |
| MANBA | 1.1241 | 8.70E-06 |  |
| HDAC2 | 1.1307 | 5.73E-03 |  |
| CLPB | 1.1347 | 4.35E-04 |  |
| GPTC8 | 1.1352 | 7.20E-03 |  |
| CIAO1 | 1.1378 | 1.17E-04 |  |
| MIC26 | 1.141 | 8.36E-04 |  |
| TPST2 | 1.1474 | 1.06E-03 |  |
| KLRB1 | 1.1497 | 3.46E-02 |  |
| TMEDA | 1.1551 | 3.89E-10 |  |
| TMED1 | 1.1614 | 1.94E-02 |  |
| CREB1 | 1.1658 | 4.06E-06 |  |
| GDAP2 | 1.1667 | 2.85E-02 |  |
| GOG8O | 1.1709 | 1.90E-02 |  |
| FND3A | 1.1751 | 4.62E-02 |  |
| NLRX1 | 1.1762 | 7.59E-03 |  |
| TPC2L | 1.1781 | 9.10E-05 |  |
| GBG11 | 1.1796 | 4.65E-02 |  |
| BCL2 | 1.181 | 1.49E-03 |  |
| MOT4 | 1.1825 | 2.63E-02 |  |
| LARP7 | 1.1825 | 2.75E-02 |  |
| CTBL1 | 1.1885 | 5.50E-08 |  |
| LAMP2 | 1.1901 | 7.38E-04 |  |
| RS9 | 1.1941 | 1.15E-02 |  |
| SMAD2 | 1.1944 | 1.29E-03 |  |
| FXR1 | 1.1973 | 3.38E-03 |  |
| PLCH1 | 1.1998 | 5.59E-04 |  |
| GT251 | 1.2096 | 1.57E-02 |  |
| MCM4 | 1.2124 | 2.84E-03 |  |
| THTM | 1.2158 | 4.21E-06 |  |
| AKP13 | 1.2174 | 1.08E-05 |  |
| TPM4 | 1.2269 | 1.03E-02 |  |
| ABC3F | 1.2276 | 4.83E-04 |  |
| PSB4 | 1.2333 | 1.14E-04 |  |
| ERLN2 | 1.235 | 6.10E-04 |  |
| KHDR1 | 1.2362 | 3.98E-05 |  |
| STT3B | 1.2536 | 4.57E-02 |  |
| TM9S2 | 1.2632 | 4.08E-04 |  |
| RL17 | 1.274 | 4.43E-04 |  |
| MON2 | 1.2762 | 1.03E-02 |  |
| RING2 | 1.2766 | 8.22E-06 |  |
| ITA5 | 1.2799 | 5.22E-05 |  |
| AK1C3 | 1.2802 | 1.94E-03 |  |
| COX7C | 1.2838 | 3.58E-03 |  |
| RL32 | 1.2855 | 2.07E-02 |  |
| GBG2 | 1.2862 | 5.33E-03 |  |
| WDR47 | 1.2866 | 4.41E-03 |  |
| HUWE1 | 1.2916 | 5.21E-06 |  |
| DIAC | 1.2966 | 2.25E-03 |  |
| NU4M | 1.2968 | 2.16E-02 |  |
| IMPA3 | 1.3046 | 1.01E-03 |  |
| SGTB | 1.3095 | 2.23E-02 |  |
| CUL4A | 1.3108 | 5.44E-06 |  |
| CCG6 | 1.3152 | 1.15E-08 |  |
| CD37 | 1.3169 | 2.26E-03 |  |
| IDE | 1.3208 | 6.17E-04 |  |
| ALG2 | 1.3235 | 2.01E-03 |  |
| S15A4 | 1.3253 | 2.83E-04 |  |
| Protein  name | **log2(FC)** | **p-value** |  |
| RM40 | 1.3274 | 9.28E-04 |  |
| ELOC | 1.34 | 6.47E-06 |  |
| IPO7 | 1.3446 | 5.76E-04 |  |
| BI1 | 1.3487 | 1.08E-02 |  |
| ABRX2 | 1.3509 | 7.93E-04 |  |
| SELH | 1.3513 | 8.94E-10 |  |
| ATG7 | 1.366 | 4.06E-02 |  |
| IQGA1 | 1.3685 | 2.98E-06 |  |
| PCAT2 | 1.3831 | 4.52E-02 |  |
| CD244 | 1.385 | 4.68E-03 |  |
| CYTSB | 1.3854 | 1.30E-02 |  |
| TMA7B | 1.392 | 3.14E-06 |  |
| PAFA2 | 1.3962 | 7.67E-03 |  |
| HSF1 | 1.3986 | 2.34E-02 |  |
| EIF3I | 1.4048 | 4.58E-11 |  |
| STRP1 | 1.4067 | 7.58E-05 |  |
| PEA15 | 1.4097 | 9.92E-03 |  |
| WBP2 | 1.4307 | 1.93E-02 |  |
| SNAB | 1.4322 | 4.68E-02 |  |
| FBX2 | 1.4397 | 1.86E-02 |  |
| PPR21 | 1.4567 | 8.22E-08 |  |
| S2546 | 1.465 | 7.52E-03 |  |
| TBCE | 1.4859 | 2.93E-05 |  |
| BL1S3 | 1.4883 | 3.05E-05 |  |
| ELOB | 1.4898 | 1.31E-05 |  |
| PEPL1 | 1.4919 | 2.21E-04 |  |
| OXR1 | 1.4972 | 3.05E-03 |  |
| GOLI4 | 1.4993 | 1.66E-02 |  |
| UBR1 | 1.5046 | 2.06E-07 |  |
| HECD1 | 1.5061 | 4.36E-04 |  |
| TSP2 | 1.5145 | 2.99E-02 |  |
| SQSTM | 1.5181 | 2.77E-02 |  |
| UBP10 | 1.5233 | 2.02E-03 |  |
| LG3BP | 1.5246 | 4.19E-02 |  |
| ERCC5 | 1.5277 | 2.80E-08 |  |
| BPHL | 1.5291 | 1.30E-02 |  |
| NFRKB | 1.5317 | 5.19E-03 |  |
| TM165 | 1.5352 | 2.45E-02 |  |
| VP33B | 1.5487 | 6.22E-05 |  |
| RT11 | 1.5547 | 3.50E-02 |  |
| SYYM | 1.5638 | 7.02E-04 |  |
| RL36A | 1.5684 | 2.01E-03 |  |
| PLCC | 1.5723 | 7.58E-03 |  |
| SEC63 | 1.5742 | 3.36E-05 |  |
| CYB | 1.5784 | 8.01E-03 |  |
| RS15A | 1.5799 | 1.48E-04 |  |
| RFA3 | 1.5819 | 8.13E-06 |  |
| NDUA9 | 1.5905 | 5.23E-04 |  |
| NUDT3 | 1.6122 | 2.62E-03 |  |
| CHM2A | 1.622 | 1.26E-04 |  |
| MSRA | 1.6318 | 6.90E-06 |  |
| PTN12 | 1.6395 | 2.95E-06 |  |
| PLXB2 | 1.6406 | 4.07E-02 |  |
| DDX41 | 1.6488 | 2.73E-05 |  |
| LACTB | 1.6609 | 2.52E-08 |  |
| TFEB | 1.6849 | 2.31E-03 |  |
| RAB3D | 1.6883 | 4.95E-02 |  |
| NAT10 | 1.6982 | 8.78E-08 |  |
| SMCA4 | 1.7257 | 3.76E-02 |  |
| AKT2 | 1.7307 | 7.53E-07 |  |
| DNJC8 | 1.7609 | 4.16E-07 |  |
| RS2 | 1.7794 | 5.96E-04 |  |
| PININ | 1.7813 | 5.26E-05 |  |
| MK08 | 1.7845 | 2.20E-02 |  |
| EIF3H | 1.7873 | 2.03E-12 |  |
| C1TM | 1.7886 | 2.09E-02 |  |
| UCK2 | 1.7895 | 1.23E-02 |  |
| 2ABD | 1.8105 | 3.60E-03 |  |
| REEP5 | 1.8112 | 7.56E-03 |  |
| Protein  name | **log2(FC)** | **p-value** |  |
| GRAA | 1.8361 | 5.82E-04 |  |
| ITA2 | 1.8429 | 3.03E-02 |  |
| NCEH1 | 1.8733 | 6.90E-05 |  |
| RBGP1 | 1.9129 | 2.88E-05 |  |
| HIP1 | 1.9454 | 4.46E-04 |  |
| VATD | 1.9581 | 1.55E-03 |  |
| KC1A | 1.9613 | 1.87E-02 |  |
| ARPC2 | 1.9769 | 1.58E-09 |  |
| UCRI | 1.9957 | 2.31E-10 |  |
| QCR1 | 2.0546 | 5.45E-10 |  |
| RL27 | 2.1636 | 2.85E-04 |  |
| RS26 | 2.1717 | 2.93E-03 |  |
| IAH1 | 2.1995 | 1.96E-08 |  |
| SUMF2 | 2.2032 | 5.31E-05 |  |
| NDUS6 | 2.2039 | 4.56E-08 |  |
| FKB15 | 2.2353 | 4.32E-07 |  |
| ANT3 | 2.2516 | 3.44E-02 |  |
| TBCC | 2.2744 | 1.78E-03 |  |
| PKRI1 | 2.3004 | 2.71E-06 |  |
| ABHDA | 2.3369 | 2.05E-09 |  |
| KBRS2 | 2.3837 | 1.20E-02 |  |
| RL37A | 2.4008 | 5.35E-04 |  |
| IGHG3 | 2.4197 | 4.94E-02 |  |
| CKAP4 | 2.4684 | 2.87E-02 |  |
| FYN | 2.475 | 2.61E-06 |  |
| CHIT1 | 2.5551 | 1.97E-10 |  |
| SNR40 | 2.5677 | 2.70E-07 |  |
| CEAM8 | 2.6044 | 5.79E-09 |  |
| RAB44 | 2.6214 | 2.36E-07 |  |
| TCPW | 2.6372 | 8.79E-10 |  |
| OLFM4 | 2.6417 | 1.20E-11 |  |
| PDIA2 | 2.6565 | 6.73E-08 |  |
| TCO1 | 2.6657 | 3.37E-10 |  |
| K2C71 | 2.6712 | 4.99E-08 |  |
| STAR9 | 2.7224 | 1.02E-08 |  |
| K2C80 | 2.7522 | 1.27E-08 |  |
| EFCB5 | 2.7723 | 9.46E-07 |  |
| CND1 | 2.7888 | 5.14E-08 |  |
| TRM61 | 2.8092 | 9.58E-06 |  |
| CRIS3 | 2.8235 | 1.42E-07 |  |
| CIA2A | 2.8411 | 2.38E-05 |  |
| CEAM1 | 2.8583 | 1.91E-07 |  |
| DLGP3 | 2.8654 | 5.97E-09 |  |
| AMPD3 | 2.8733 | 3.94E-05 |  |
| DQB1 | 2.8749 | 2.27E-08 |  |
| MSI1H | 2.8798 | 8.38E-09 |  |
| RM21 | 2.8838 | 1.42E-04 |  |
| NEUL | 2.8905 | 4.74E-04 |  |
| DYR | 2.8975 | 6.80E-08 |  |
| CE170 | 2.9356 | 1.53E-04 |  |
| PREX1 | 2.9418 | 3.45E-10 |  |
| SCMC2 | 2.9424 | 6.21E-07 |  |
| SVIL | 2.9476 | 2.43E-04 |  |
| GOLI | 2.9634 | 4.25E-06 |  |
| CL12A | 2.9791 | 3.86E-05 |  |
| FADD | 2.9939 | 5.98E-09 |  |
| RM10 | 3.03 | 1.80E-06 |  |
| CLPX | 3.037 | 8.96E-10 |  |
| OS9 | 3.0421 | 2.34E-05 |  |
| NBAS | 3.0531 | 1.55E-05 |  |
| SMTN | 3.0535 | 2.43E-03 |  |
| GRAK | 3.0576 | 4.31E-03 |  |
| SCFD2 | 3.1195 | 1.46E-04 |  |
| SIGL5 | 3.1442 | 1.18E-02 |  |
| MAGD2 | 3.1629 | 9.54E-03 |  |
| DPOD1 | 3.1748 | 7.00E-04 |  |
| RRP12 | 3.1782 | 9.41E-04 |  |
| GCYB1 | 3.1788 | 2.03E-02 |  |
| T132B | 3.2082 | 3.96E-05 |  |

| Protein  name | log2(FC) | p-value |
| --- | --- | --- |
| SPB12 | 3.2128 | 9.06E-04 |
| RSBNL | 3.2136 | 2.66E-03 |
| PLD4 | 3.2166 | 3.15E-03 |
| TMC5B | 3.2169 | 9.40E-04 |
| TM214 | 3.2217 | 1.14E-03 |
| STAB1 | 3.2331 | 1.76E-03 |
| MTND | 3.2358 | 7.98E-06 |
| CO7 | 3.2376 | 4.74E-04 |
| P121C | 3.2414 | 2.71E-05 |
| CD6 | 3.2689 | 2.43E-05 |
| ANO10 | 3.2714 | 3.65E-04 |
| NLRC4 | 3.3051 | 2.18E-03 |
| RAB38 | 3.3057 | 4.16E-03 |
| MAFG | 3.3117 | 4.58E-07 |
| RT63 | 3.3243 | 1.52E-03 |
| CTDSL | 3.3313 | 4.42E-02 |
| DOT1L | 3.3749 | 2.06E-06 |
| SLIK5 | 3.3951 | 8.98E-06 |
| SUMF1 | 3.4099 | 3.14E-03 |
| TFPT | 3.4125 | 1.22E-03 |
| NID2 | 3.4165 | 1.92E-03 |
| CTNA1 | 3.4198 | 3.01E-07 |
| STK3 | 3.4205 | 6.79E-04 |
| PGH2 | 3.4371 | 2.71E-02 |
| C163A | 3.482 | 3.20E-02 |
| KRT86 | 3.4824 | 3.13E-02 |
| CAP7 | 3.4887 | 1.33E-02 |
| TBB6 | 3.494 | 3.42E-06 |
| IGFR1 | 3.4984 | 9.97E-05 |
| AL7A1 | 3.5131 | 1.01E-02 |
| PIGR | 3.5143 | 1.10E-02 |
| SORT | 3.5188 | 6.09E-03 |
| LRRC2 | 3.5246 | 3.47E-05 |
| NHLC3 | 3.5328 | 1.09E-06 |
| SMC2 | 3.5356 | 8.67E-03 |
| MTMRE | 3.542 | 5.08E-03 |
| WIPI4 | 3.5585 | 3.31E-04 |
| MIEAP | 3.5746 | 6.31E-05 |
| NMT2 | 3.5754 | 5.69E-03 |
| TNAP2 | 3.5857 | 4.70E-02 |
| DNJC5 | 3.6008 | 1.53E-04 |
| ENOG | 3.6065 | 1.30E-06 |
| PAI1 | 3.6511 | 8.33E-03 |
| RGPA2 | 3.6746 | 2.72E-03 |
| RN167 | 3.6917 | 1.18E-04 |
| 4EBP1 | 3.6959 | 2.09E-02 |
| CD109 | 3.7229 | 2.04E-03 |
| TBA4B | 3.7556 | 1.69E-06 |
| DYN1 | 3.7639 | 1.61E-05 |
| CPNE2 | 3.7718 | 3.15E-02 |
| PPR3D | 3.7777 | 1.14E-02 |
| EGF | 3.7891 | 2.52E-02 |
| PLOD1 | 3.8043 | 2.34E-04 |
| CE152 | 3.8138 | 2.61E-03 |
| RM33 | 3.8203 | 6.06E-03 |
| Protein  name | **log2(FC)** | **p-value** |
| DEGS1 | 3.8255 | 9.77E-04 |
| COXM1 | 3.8296 | 5.11E-03 |
| ARMX3 | 3.8629 | 4.07E-03 |
| APOL1 | 3.8752 | 1.12E-10 |
| BMX | 3.8891 | 7.60E-03 |
| ATG4B | 3.9516 | 8.25E-05 |
| SMRC1 | 3.99 | 1.07E-05 |
| SYTL4 | 4.0214 | 2.19E-02 |
| CP4V2 | 4.0254 | 1.31E-04 |
| HIDE1 | 4.0303 | 4.87E-03 |
| FOS | 4.0306 | 2.73E-03 |
| DRS7B | 4.032 | 3.89E-03 |
| ABCD1 | 4.1494 | 1.02E-02 |
| LRC8D | 4.1618 | 1.58E-02 |
| GPC4 | 4.1737 | 4.55E-05 |
| DIAP2 | 4.2243 | 9.21E-03 |
| UNG | 4.2328 | 4.56E-04 |
| AN34C | 4.2746 | 9.94E-03 |
| ATLA1 | 4.2827 | 3.53E-03 |
| ZN586 | 4.3326 | 2.31E-03 |
| VAMP3 | 4.3615 | 2.53E-03 |
| EMC8 | 4.4004 | 4.73E-04 |
| ATRIP | 4.4088 | 3.03E-03 |
| IGSF2 | 4.4776 | 8.57E-03 |
| LAMA1 | 4.4957 | 1.27E-02 |
| ACACA | 4.6438 | 4.08E-03 |
| AT11B | 4.6763 | 2.07E-02 |
| OFD1 | 4.7104 | 4.30E-02 |
| K2C78 | 4.7312 | 7.45E-12 |
| BMP2 | 4.7859 | 3.97E-03 |
| PTMS | 4.7929 | 3.46E-02 |
| ING3 | 4.8067 | 6.54E-03 |
| S39A7 | 4.8073 | 7.79E-04 |
| WDFY4 | 4.8542 | 4.71E-03 |
| M3K5 | 4.8827 | 2.66E-06 |
| WBP1L | 4.9814 | 6.68E-03 |
| RNAS6 | 5.1182 | 4.58E-02 |
| IL2RG | 5.1942 | 6.47E-04 |
| MB214 | 5.229 | 5.09E-03 |
| AT2L2 | 5.2668 | 2.44E-05 |
| OR1M1 | 5.3051 | 1.86E-04 |
| BL1S2 | 5.4157 | 3.70E-06 |
| DYH7 | 5.5075 | 3.34E-04 |
| CLCC1 | 5.748 | 4.27E-03 |
| GCP4 | 5.7589 | 9.07E-03 |
| CEBPB | 5.87 | 7.51E-03 |
| FA76B | 6.1636 | 6.53E-05 |
| RT06 | 6.4839 | 6.89E-04 |
| KKCC2 | 6.4923 | 2.02E-02 |
| FNIP2 | 6.5274 | 1.30E-05 |
| RAB35 | 6.6834 | 3.92E-06 |
| CPT2 | 7.3339 | 6.91E-09 |
| F131A | 7.5586 | 5.31E-05 |
| TRY2 | 7.9747 | 2.68E-04 |
| PMF1 | 8.2266 | 1.70E-05 |

**Supplementary Table 4**. List of upregulated pathways in NR patients using Hallmarks database (significant when FDR < 0.25).

| Pathway | Size | ES | NES | NOM p-value | FDR | RANK AT MAX | LEADING EDGE |
| --- | --- | --- | --- | --- | --- | --- | --- |
| *TGF_BETA_SIGNALING* | 20 | 0.500 | 0.500 | 0.090 | 0.030 | 528 | tags=35%, list=11%, signal=39% |
| *TNFA_SIGNALING_VIA_NFKB* | 45 | 0.516 | 0.516 | 0.004 | 0.045 | 1098 | tags=42%, list=23%, signal=54% |
| *SPERMATOGENESIS* | 22 | 0.449 | 0.449 | 0.050 | 0.075 | 75 | tags=14%, list=2%, signal=14% |
| *P53_PATHWAY* | 68 | 0.391 | 0.391 | 0.030 | 0.167 | 596 | tags=24%, list=12%, signal=26% |
| *KRAS_SIGNALING_UP* | 56 | 0.392 | 0.392 | 0.065 | 0.204 | 657 | tags=21%, list=14%, signal=25% |
| *PEROXISOME* | 52 | 0.365 | 0.365 | 0.081 | 0.213 | 893 | tags=25%, list=19%, signal=30% |
| *UV_RESPONSE_DN* | 46 | 0.369 | 0.369 | 0.076 | 0.231 | 905 | tags=26%, list=19%, signal=32% |
