# Supplementary material for: Imbalanced NK cell subpopulations and TIGIT expression limit cetuximab efficacy in colorectal cancer: A promising target for treatment enhancement
Source: Clin Transl Med. 2025 Jun 9;15(6):e70351. doi: 10.1002/ctm2.70351 (PMC12148951; doi:10.1002/ctm2.70351)
Supplement: Supplementary file 3 — Supporting Information [file CTM2-15-e70351-s003.docx]

**Appendix 3: Supplementary Figures**

**Supplementary Figure 1.** Overall survival (OS) and time to progression (TTP) rates according to the response to cetuximab treatment in mCRC patients. Kaplan-Meir curves are shown for non-responders (NR) and responders (R) patients. Log rank (Mantel-Cox) method was used for statistical analysis. Hazard Ratio (HR) was obtained using NR as reference.

**Supplementary Figure 2.** CD45+ lymphocytes found in mCRC patients. Red: NK cells; orange: NKT-like cells; green: T cells.


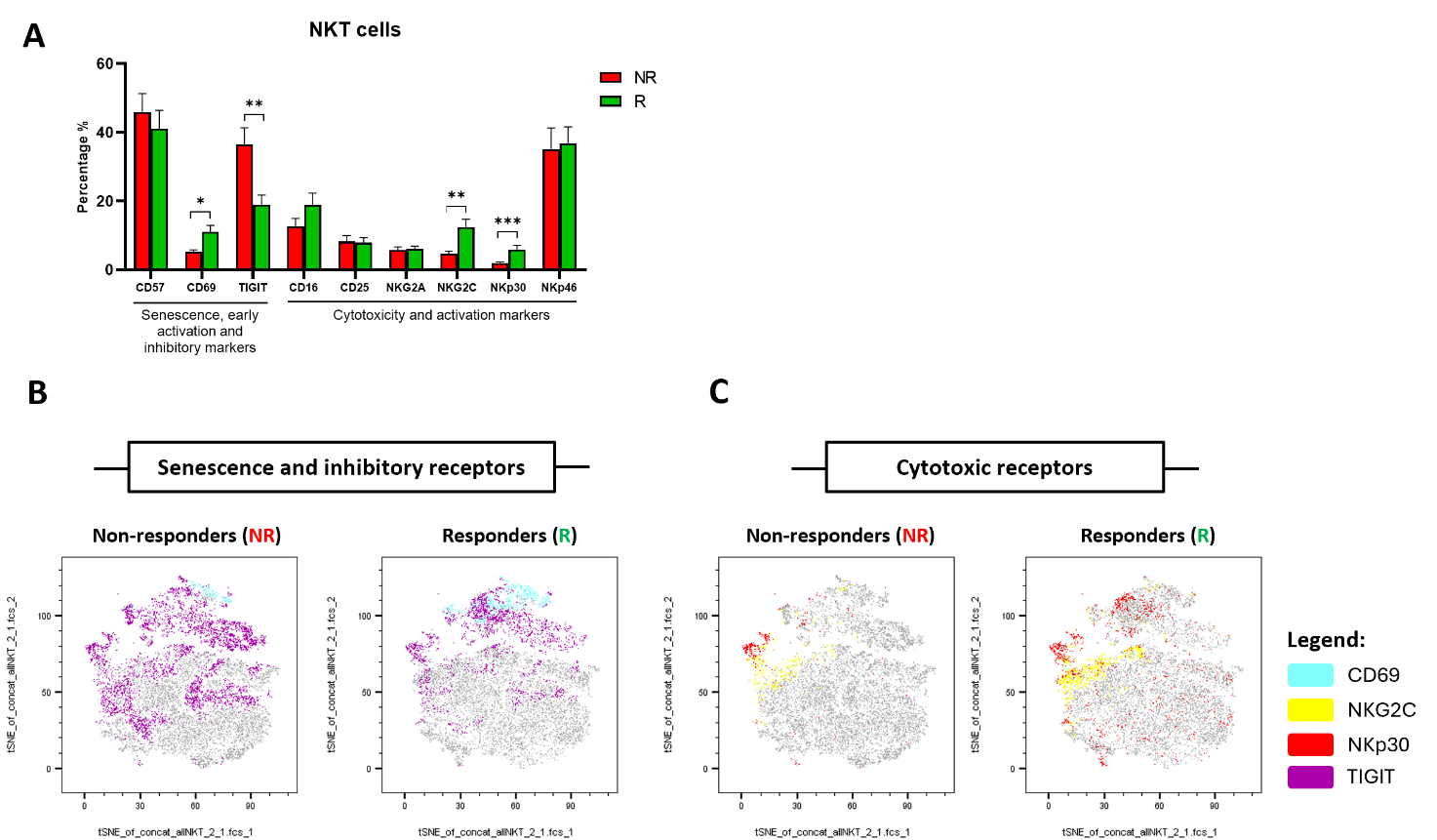


**Supplementary Figure 3.** Analysed markers in NKT cells. **(A)** Bar graph showing the most significative markers. **(B)** tSNE plot of significant senescence and inhibitory receptors **(C)** and cytotoxic receptors in NKT cells. *: p < 0.05; **: p < 0.01; ***: p < 0.001; ****: p < 0.0001.


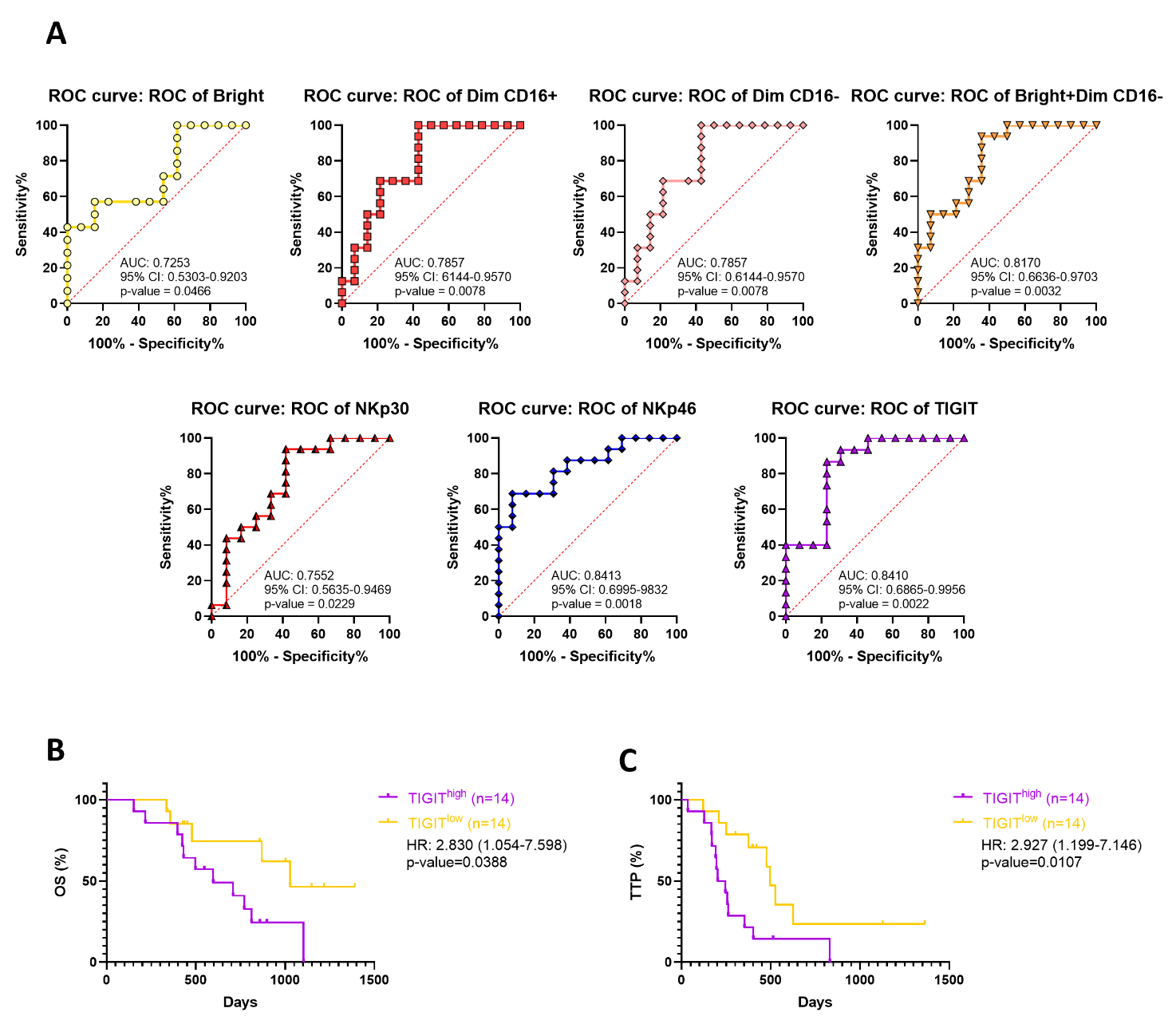


**Supplementary Figure 4. (A)** ROC curves of the most significant parameters analysed by flow cytometry in mCRC patients. AUC: Area Under Curve; 95% IC: 95% Interval of Confidence. **(B)** Prognostic value of TIGIT for the survival risk stratification of mCRC patients receiving cetuximab. The median expression of TIGIT in NK cells (17.75%) was used as a cut-off and the corresponding Kaplan-Meier curves for overall survival (OS) and **(C)** time to progression (TTP) rates are shown. Hazard Ratio (HR) was obtained using TIGIT^high^ as reference.
